# Wild Egyptian medicinal plants show in vitro and in vivo cytotoxicity and antimalarial activities

**DOI:** 10.1186/s12906-022-03566-5

**Published:** 2022-05-12

**Authors:** Ahmed M. Abdou, Abdel-latif S. Seddek, Noha Abdelmageed, Mohamed O. Badry, Yoshifumi Nishikawa

**Affiliations:** 1 National Research Center for Protozoan Diseases, Obihiro University of Agriculture and Veterinary Medicine, Inada-Cho, Obihiro, Hokkaido 080-08555 Japan; 2 Department of Forensic Medicine and Toxicology, Faculty of Veterinary Medicine, South Valley University, Qena, 83523 Egypt; 3 Department of Pharmacology, Faculty of Veterinary Medicine, Sohag University, Sohag, 82524 Egypt; 4 Department of Botany and Microbiology, Faculty of Science, South Valley University, Qena, 83523 Egypt

**Keywords:** Egypt, Desert, Malaria, Parasitemia, *Plasmodium falciparum*, *Plasmodium yoelii*, Mice

## Abstract

**Background:**

Medicinal plants have been successfully used as an alternative source of drugs for the treatment of microbial diseases. Finding a novel treatment for malaria is still challenging, and various extracts from different wild desert plants have been reported to have multiple medicinal uses for human public health, this study evaluated the antimalarial efficacy of several Egyptian plant extracts.

**Methods:**

We assessed the cytotoxic potential of 13 plant extracts and their abilities to inhibit the in vitro growth of *Plasmodium falciparum* (3D7), and to treat infection with non-lethal *Plasmodium yoelii* 17XNL in an in vivo malaria model in BALB/c mice.

**Results:**

In vitro screening identified four promising candidates, *Trichodesma africanum, Artemisia judaica, Cleome droserifolia*, and *Vachellia tortilis*, with weak-to-moderate activity against *P. falciparum* erythrocytic blood stages with mean half-maximal inhibitory concentration 50 (IC_50_) of 11.7 μg/ml, 20.0 μg/ml, 32.1 μg/ml, and 40.0 μg/ml, respectively. Their selectivity index values were 35.2, 15.8, 11.5, and 13.8, respectively. Among these four candidates, *T. africanum* crude extract exhibited the highest parasite suppression in a murine malaria model against *P. yoelii*.

**Conclusion:**

Our study identified novel natural antimalarial agents of plant origin that have potential for development into therapeutics for treating malaria.

**Supplementary Information:**

The online version contains supplementary material available at 10.1186/s12906-022-03566-5.

## Background

Malaria is caused by parasites belonging to the phylum Apicomplexa, genus *Plasmodium*. In 2019, approximately 229 million cases of malaria and 409,000 associated deaths were reported across 87 malaria-endemic countries [1]. Human malaria cases are caused by four different *Plasmodium* species*—Plasmodium ovale*, *Plasmodium vivax*, *Plasmodium malariae, Plasmodium falciparum—*of which *P. falciparum* is considered to be the most lethal [2, 3]. *Plasmodium* parasites are typically transmitted by the bite of an infected female *Anopheles* mosquito, although malaria can also be transmitted through exposure to blood products from an infected individual (transfusion malaria) or congenitally [4]. Multidrug-resistant *Plasmodium* parasites are the biggest challenge to health care in most malaria-endemic areas. Thus, research to develop new antimalarial drugs is critical [5].

The two main malaria species that are responsible for most human malaria cases, *P. falciparum* and *P. vivax*, have developed resistance against chloroquine. This drug resistance first emerged in the late 1950s in both Colombia and at the Cambodia–Thailand border and may stem from the great success of chloroquine and its multiple large-scale usages over the decades [6]. Great efforts have been made to develop the novel active agent, artemisinin, as an alternative drug to chloroquine. However, presently, there is no single drug effective for treating multi-drug resistant malaria, and effective combination treatment includes artemisinin derivatives, such as artesunate, or mixtures with previously developed drugs, such as an atovaquone-proguanil combination [7].

From ancient times, medical plants have been used for various pharmacological purposes because they contain many useful biological compounds [8]. More than 1277 plant species have been traditionally used for the treatment of malaria [9, 10]. Natural products still have an effective role in disease treatment. Finding anti-parasitic compounds produced by natural products, especially traditional medicinal plants from Asia, Africa, or the Americas, which have been reported as being successfully used to treat many diseases, could be an initial step toward controlling an array of diseases [11]. Currently, the WHO recommends widespread use of the RTS,S/AS01 (RTS,S) malaria vaccine among children at risk in sub-Saharan Africa [12]. Egypt has multiple aromatic and medicinal plants owing to its favorable geographical position, climate, and soil condition; thus, it is a useful site for exploring herbal and medicinal plants [13]. Previous studies illustrated the use of plant extracts in inhibition of *P. falciparum* in vitro [14–17] and in vivo using various doses of *Ficus platyphylla* plant extract ranging from 100 to 300 mg/kg/day against *Plasmodium berghei* infection [18]. In addition, other studies evaluated the effect of plant extracts by oral treatment in a murine model by chemotherapeutic test against different murine *Plasmodium* species in BALB/c mice [19–21]. Furthermore, the combination of the plant extracts with the reference drug artemisinin in treatment of *Plasmodium yoelii* was also reported [22]. Other reported study evaluated febrifugine and isofebrifugine mixture prepared from the dried leaves of *H. macrophylla var. Otaksa* against three rodent *Plasmodia* species; *P. yoelii* 17XL, *P. berghei* NK65, and *P. chabaudi* AS in Institute of Cancer Research (ICR) mice [23]. The previous reported experimental models support the use of our in vitro assay and in vivo model in treatment of *P. falciparum* in vitro and *P. yoelii*-infected mice in murine malaria model. Therefore, the present study aimed to evaluate the effectiveness of extracts from Egyptian medicinal plants randomly selected from the desert roads against human malaria, first via an in vitro assay and then with a murine malaria model.

## Methods

### Ethical statement

This study was performed in strict accordance with the recommendations of the Guide for the Care and Use of Laboratory Animals of the Ministry of Education, Culture, Sports, Science and Technology, Japan. The protocol was approved by the Committee on the Ethics of Animal Experiments at Obihiro University of Agriculture and Veterinary Medicine, Obihiro, Japan (permit numbers 19–185, 20–157, 21–32). Mouse work, such as injection with parasites or extracts, and euthanasia was implemented under general inhalation anesthesia induced with isoflurane (2%) to minimize animal suffering. Mice were euthanized by cervical dislocation at 30 days after parasite infection.

### *P. Falciparum* culture and maintenance

*Plasmodium falciparum* parasites were transferred to previously washed human O^+^ red blood cells (RBCs) obtained from Hokkaido Red Cross Blood Center maintained in fresh complete RPMI-1640 medium (Sigma, St Louis, MO, USA), which was supplemented with a mixture of 6 g of HEPES (Sigma), 2 g of NaHCO_3_, 25 mg of hypoxanthine, 5 g of albumax II (Gibco, Carlsbad, CA, USA), and 250 μl of gentamicin (stock concentration, 50 mg/ml) in dissolved in MilliQ water. The final prepared complete medium was filtered with a 0.20-μm membrane filter (IWAKI, Saitama, Japan). The parasite cultures were maintained at 37 °C in a 5% CO_2_ atmosphere.

### Plant material collection and extraction

The plants used in this study were obtained from a field survey conducted in two locations in Qena governorate (Latitude: 26° 09′ 51.05“ N, Longitude: 32° 43’ 36.16” E), which is in the southern region of Egypt: Qena-Sohag and Qena-Safaga (after Km 85) desert roads, Eastern desert, Egypt. A map marking the collection sites is shown in Fig. S1. Plants collection sites coordinates were shown in Table S1. Material was collected from 13 different plant species in May 2019 (plant flowering season). Samples were collected between 4:00 AM and 12:00 PM. The plant samples were collected under the approval of South Valley University, Qena, Egypt and were microscopically identified by Dr. Mohamed Owis Badry, in the herbarium of South Valley University at Faculty of Science, South Valley University, Egypt and voucher specimens were deposited in the same herbarium. Identification was performed according to the available literature [24–27], and an official identification letter was obtained. Images of the herbarium sheets of the identified plant species from which samples were collected are shown in Fig. S2. Plant taxonomy and species were further updated in accordance with information from Plants of the World Online [28].

For plant collection from the study areas, although that there are no specific licenses were required for the field studies, permission for collection of plants was obtained from Faculty of Veterinary Medicine, South Valley University, Qena, Egypt. Collection was performed under the guidelines and rules of South Valley University, Qena. The surveyed locations were not protected or privately-owned in any way and the field studies did not include any protected or endangered Egyptian plant species. The Latin binomial names of all plant extracts studied in this study were shown in Table 1.

**Table 1:** Latin binomial name of all plant extracts used in this study

Plant Extract	Family	Latin binomial name
*Aerva javanica*	Amaranthaceae	*Aerva javanica* (Burm.f.) Juss. ex Schult.
*Anabasis setifera*	Amaranthaceae	Anabasis setifera Moq.
*Artemisia judaica*	Asteraceae	Artemisia judaica L.
*Calotropis procera*	Apocynaceae	*Calotropis procera* (Aiton) W.T.Aiton
*Carthamus tinctorius*	Asteraceae	*Carthamus tinctorius* L.
*Citrullus colocynthis*	Cucurbitaceae	*Citrullus colocynthis* (L.) Schrad.
*Cleome droserifolia*	Cleomaceae	Cleome droserifolia (Forssk.) Delile
*Forsskaolea tenacissima*	Urticaceae	Forsskaolea tenacissima L.
*Ochradenus baccatus*	Resedaceae	Ochradenus baccatus Delile
*Ocimum basilicum*	Lamiaceae	*Ocimum basilicum* L.
*Pulicaria undulata*	Asteraceae	Pulicaria undulata (L.) C.A.Mey.
*Trichodesma africanum*	Boraginaceae	Trichodesma africanum (L.) Sm.
*Vachellia tortilis*	Fabaceae	*Vachellia tortilis* subsp. raddiana (Savi) Kyal. & Boatwr.

Plants used in this study was collected from the wild survey from the desert roads around Qena Governorate and were identified microscopically in South Valley University herbarium, Faculty of science, South Valley university, Qena, Egypt. Latin names were provided in the identification letter

Plant samples were dried in the shade for 3–10 days, then a fine powder was obtained from the dried leaves, flowers, fruit, or seed parts by using a kitchen blender. The powdered plant material from each plant was dissolved at a 1:10 ratio in 80% methanol, 70% ethanol, or distilled water (100 g of plant powder/1 L of solvent) for a minimum of 1–3 days. The plant supernatant was further collected and filtrated by glass filtration apparatus and was collected in wide conical flask, and then it was dissolved in a wide petri dish at room temperature for 1–3 days. The final crude extract was collected in centrifuge tubes and stored in − 30 °C until use. To test the antimalarial potential of the various plant extracts, they were solubilized individually in the solvent dimethyl sulfoxide (DMSO) to prepare stock solutions (100 mg/ml).

### Determination of cytotoxicity of plant extracts

To determine the cytotoxic potential of the plant extracts, their cytotoxicity against human foreskin fibroblast (HFF) cells was evaluated. Cell suspensions (1 × 10^5^ cells/ml) in Dulbecco’s Modified Eagle medium (DMEM, Sigma-Aldrich, St. Louis, MO, USA) supplemented with 10% fetal bovine serum (FBS) (Nichirei Bioscience, Tokyo, Japan) were plated at 100 μl/well in 96-well plates and incubated at 37 °C in a 5% CO_2_ atmosphere for 48 h. The plant extracts were added to the cells at final concentrations of a two-fold serial dilution starting from 1000 μg/ml. To evaluate cell viability, cell proliferation inhibition (%) was calculated as described previously [29, 30].

### In vitro anti-plasmodial activity

*Plasmodium falciparum* (3D7 strain) was maintained in O^+^ human erythrocytes (1% hematocrit) in complete RPMI medium (Sigma-Aldrich). *P. falciparum* was further synchronized to the ring stage with 5% sorbitol (>90%, as verified by light microscopy on Giemsa-stained blood smears [Giemsa stain for microscopy, Merck, Darmstadt, Germany]). Parasite solutions were prepared at 0.5% parasitemia and 2% hematocrit in complete RPMI medium. A 50-μl sample of the infected erythrocytes was added to each well of 96-well plates containing 50 μl of plant extract (concentrations ranging from 0.25–100 μg/ml). Medium only was used as a negative control, while chloroquine was used as a positive control. The plates were then incubated in an atmosphere of 5% CO_2_, 5% O_2_ at 37 °C for 72 h.

Parasite growth inhibition was determined by adding 100 μl of 0.02% of Syber Green I stain (SYBR® Green I Nucleic acid stain 10,000×, Lonza, Rockland, ME, USA) in lysis buffer (25 mM Tris, pH 7.5, containing 10 mM ethylenediamine tetraacetic acid, 0.01% saponin, and 0.1% Triton X-100) to each well, mixing gently, and incubating the plates for 1–2 h in the dark [31, 32]. The relative fluorescent inhibition values were determined by using a fluorescent plate reader Fluoroskan Ascent (Thermo Labsystems, Waltham, MA, USA) with excitation and emission wavelengths of 485 nm and 518 nm, respectively [29, 31, 33]. Parasite morphology was observed by examining Giemsa-stained blood smears with an all-in-one microscope BioRevo BZ-9000 (Keyence BioRevo, Tokyo, Japan). Parasite growth inhibition percentages were calculated as described previously [31, 33]. The antiplasmodial activities of the natural plant extracts used in this study were classified as follows: IC_50_ < 0.1 μg/ml: very good activity; IC_50_ between 0.1–1 μg/ml: good activity; IC_50_ between 1.1–10 μg/ml: good to moderate activity; IC_50_ between 11 and 50 μg/ml weak activity; IC_50_ > 100 μg/ml: inactive according to the classification mentioned in the reported study [34].

### In vivo antimalarial efficacy of plant extracts

BALB/c mice, originally purchased from Clea Japan (Tokyo, Japan), were bred under specific pathogen-free conditions in the animal facility of the National Research Center for Protozoan Diseases at Obihiro University of Agriculture and Veterinary Medicine, Obihiro, Japan. The animals were treated in accordance with the guiding principles for the care and use of research animals published by the Obihiro University of Agriculture and Veterinary Medicine, Obihiro, Japan. The animals were kept under standard laboratory conditions on a 12/12-h light/dark cycle at 21 °C under 40% relative humidity and fed with commercial food and water ad libitum.

The non-lethal strain *Plasmodium yoelii* 17XNL was recovered from a stock of frozen parasitized RBCs (pRBC) via passage in donor mice intraperitoneally inoculated. Parasitemia was monitored daily. When the parasitemia level reached 20–30%, the donor mice were anesthetized, and blood was collected by cardiac puncture into a syringe containing 0.1 ml of ethylene diamine-N, N, N′, N′- tetraacetic acid disodium salt (EDTA) (Djindo Kumamoto, Japan).

Two male BALB/c mice aged 8–10 weeks and weighing 25–30 g, were infected with approximately 1 × 10^7^*P. yoelii*-infected erythrocytes in total volume of 0.5 ml of phosphate-buffered saline (PBS). For each independent experiment, the mice were randomly divided into three groups of five according to previous published studies. AMA was aware of the group allocation at the different stages of the experiment. Total 17 mice were used for one trial. When the level of parasitemia reached 1%, oral treatment using 100 mg/kg/day of plant extracts was begun and continued for 1 week from day 0 (3 h post-challenge) until day 6 post-infection. The negative-control animals received only PBS. The parasitemia was assessed daily until 30 days post-infection by examining thin blood films made from mouse tail blood and stained with 10% Giemsa solution. The films were examined using a microscope to determine the parasite suppression activity of each extract. To measure the hematocrit percentage, 10 μl of blood was collected from the tail vein every other day until 30 days post-infection and measured by Celltac-α MEK-6550 (Nihon Kohden, Tokyo, Japan). A parasitemia suppression test (chemotherapeutic test) was performed daily for 1 week from challenge (day 0) until 6 days post-infection; the percentage of parasite growth suppression was calculated by using the following previously reported eq. [35], which is slightly modified from the study that originally reported it [36].

1 $$\%\ of\ parasite\ growth\ suppression=\left(A-B\right)/A\times 100$$

Where *A* is the mean parasitemia of the untreated group and *B* is parasitemia of each individual mouse in the treated groups.

The parasitemia percentage, bodyweight, and survival rates were monitored daily, and the hematocrit was monitored every other day. The percentage of parasitemia of each mouse was calculated by counting the number of parasite-infected erythrocytes per 600–1000 erythrocytes visible under a light microscope in 4–5 randomly selected fields of methanol-fixed thin blood smears slides stained with 10% Giemsa solution.

2 $$\%\;Parasitemia=\left(number\;of\;infected\;RBCs\right)/\left(total\;Number\;of\;RBCs\;\right)\times100$$

Order of treatment starts from the control then the treated groups. Order of challenge infection starts from the control then the treated groups. Measurements of body weight, hematocrit, and parasitemia were randomly done in group starting from control then the treated groups. Cage location was not changed from the start of the experiment until the end.

### Statistical analysis

Graph Pad Prism 8.4.3 software (Graph Pad Software Inc. La Jolla, CA, USA) was used for all statistical tests. For the in vitro data, the IC_50_ values for the inhibition percentage of parasites and host cells were determined. The final mean IC_50_ of anti-*P. falciparum* (3D7) activity was calculated based on three independent experiments, and mean IC_50_ values against HFF cells were calculated based on three independent experiments. For the in vivo data (mean parasitemia %, mouse bodyweight and hematocrit changes), statistical analyses were performed using a two-way analysis of variance (ANOVA). Survival curves were generated with the Kaplan–Meier method, and survival rates were analyzed by a χ^2^ test. Statistically significant differences (those with a *p*-value of < 0.05) are marked in the figures by asterisks and defined in each figure legend. There were no any criteria used for including and excluding animals.

## Results

### In vitro antimalarial efficacy of plant extracts

The in vitro activities against *P. falciparum* 3D7 growth of 13 different types of Egyptian plant extracts were evaluated. Among the 13 tested plant extracts, four (extracts of *Trichodesma africanum*, *Artemisia judaica*, *Cleome droserifolia,* and *Vachellia tortilis*) showed low-to-moderate activity against *P. falciparum* 3D7; their mean IC_50_ values were 11.7 μg/ml, 20.0 μg/ml, 32.1 μg/ml, and 40.0 μg/ml, respectively, and their mean selectivity index values were 35.2, 15.8, 11.5, and 13.8, respectively, (Table 2). Despite the low-to-moderate activities of the previously mentioned plant extracts, they possess good selectivity index values (Table 2). The ethanolic extract of *Pulicaria undulata* and both the ethanolic and methanolic extracts of *Citrullus colocynthis* showed weak activity against *P. falciparum* 3D7 growth (mean IC_50_ values: 18.9 μg/ml, 51.7 μg/ml, 45.9 μg/ml, respectively) and had mean selectivity index values of 2.9, 1.7, and 1.4, respectively (Table 2).

**Table 2:** Mean IC_50_ of Egyptian plant extracts against *Plasmodium falciparum* (3D7) and HFF cells in vitro

Plant Extract		Plant family	Plant part	Mean IC_50_ (μg/ml)		Mean Selectivity index (SI)
				*P. falciparum* (3D7) ^a^ (±SD)	HFF cells ^b^ (±SD)	
*Aerva javanica (Burm.f.) Juss. ex Schult.*		Amaranthaceae	leaves	> 100 (43.4)	378.1 (134.0)	> 3.7
*Anabasis setifera Moq.*		Amaranthaceae	leaves	> 100	1263.6 (194.9)	> 12.6
*Artemisia judaica L.*		Asteraceae	leaves	20.0 (3.5)	316.8 (88.8)	15.8
*Calotropis procera (Aiton) Dryand.*	*aq.*	Apocynaceae	flowers	> 100	41.5 (22.6)	> 0.4
	*M80%*		flowers	> 100	2.9 (1.5)	> 0.02
*Carthamus tinctorius L.*		Asteraceae	flowers	> 100	444.7 (169.5)	> 4.4
*Citrullus colocynthis (L.) Schrad.*	*E70%*	Cucurbitaceae	seeds	51.7 (10.8)	88.0 (14.3)	1.7
	*M80%*		seeds	45.9 (23.3)	65.6 (7.2)	1.4
*Cleome droserifolia (Forssk.) Delile*		Cleomaceae	leaves	32.1 (3.8)	370.9 (95.3)	11.5
*Forsskaolea tenacissima L.*		Urticaceae	leaves	> 100	519.0 (141.9)	> 5.1
*Ochradenus baccatus Delile*		Resedaceae	fruit	> 100	1179. 0 (245.4)	> 11.7
*Ocimum basilicum L. (E70%)*		Lamiaceae	leaves	> 100	252.6 (13.9)	> 2.5
*Pulicaria undulata (L.) C.A.Mey.*	*(E70%)*	Asteraceae	flowers	18.9 (2.8)	55.5 (10.7)	2.9
	*(M80%)*		flowers	> 100 (38.2)	197.5 (61.3)	> 1.9
*Trichodesma africanum (L.) Sm.*		Boraginaceae	leaves	11.7 (4.7)	413.0 (96.9)	35.2
*Vachellia tortilis subsp. raddiana (Savi) Kyal. & Boatwr.*		Fabaceae	seeds	40.0 (2.8)	554.5 (110.5)	13.8
Chloroquine				0.009		

^a^ The mean IC_50_ and standard deviation values against *P. falciparum* were calculated from the average of three independent experiments after a 72-h culture of the parasites with a plant extract. ^b^ the mean IC_50_ against HFF cells was calculated from three independent experiments after a 72-h culture. Except those that are indicated to have an ethanolic or aqueous extract, all the plant extractions are methanolic. *IC*_*50*_ half maximal inhibitory concentration 50, *SD* standard deviation, *SI* selectivity index, *HFF* human foreskin fibroblast, *M80* 80% methanol, *E70* 70% ethanol, *aq*. aqueous

All extracts of *Aerva javanica* and *Anabasis setifera,* both the aqueous and methanolic extracts of *Calotropis procera, Carthamus tinctorius, Forsskaolea tenacissima, Ochradenus baccatus*, and *Ocimum basilicum*, and the methanolic extract of *P. undulata* showed no efficacy against the growth of *P. falciparum* (3D7) in vitro, with IC_50_ values of > 100 μg/ml (Table 2).

### In vitro effect of plant extracts on *P. falciparum* growth stages and morphology

**Fig. 1:**
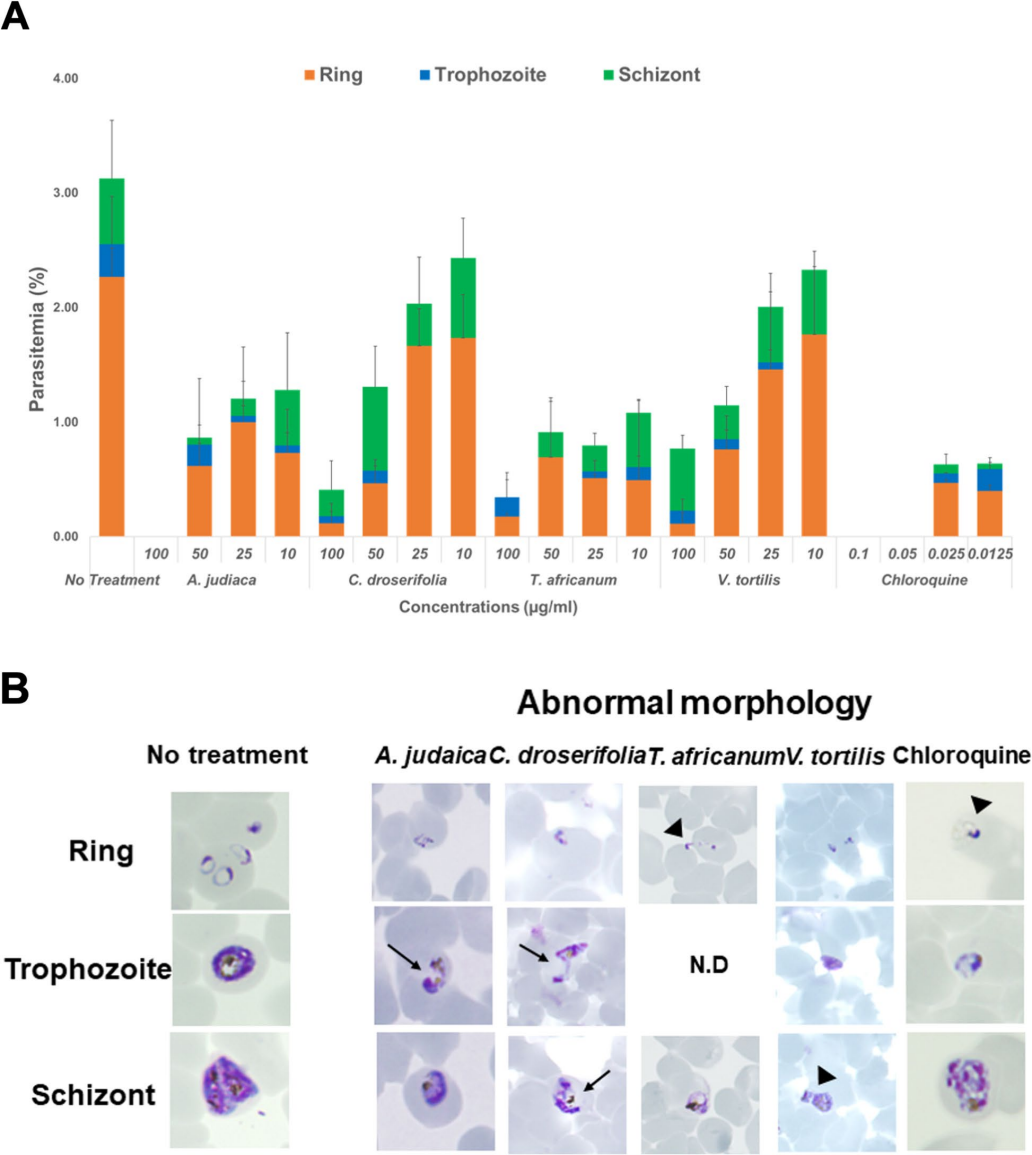
Effect of plant extracts on stage-specific *P. falciparum* (3D7) morphology in vitro. **A** Percentage of parasites at each stage (i.e., ring, trophozoite, or schizont) after treatment with 10, 25, 50, or 100 μg/ml of extracts of *Artemisia judaica, Trichodesma africanum, Cleome droserifolia*, or *Vachellia toritilis* plants, chloroquine, or medium alone. The number of parasites at each stage was determined from a total of 600–900 erythrocytes. Data are representative of two independent experiments with similar results. **B ***P. falciparum* parasites were treated with 50 μg/ml of an extract of *A. judaica*, *T. africanum, C. droserifolia*, or *V. tortilis*. Chloroquine (0.025 μg/ml) was used as a positive control, and medium alone was used as a negative control. Three wells were used for each plant or drug concentration. After 72 h, the parasite morphology was observed via microscopy (× 100 magnification) on Giemsa-stained thin blood smears. Line arrow indicate the fragmented parasites, while arrow head indicate the shrinkage parasites. Data shown here are representative of two independent experiments that produced similar results

To confirm the in vitro antimalarial efficacy of the tested plant extracts, we observed thin blood smears from 72-h parasite culture (Fig. 1). Treatment with plant extracts for 72 h caused dose-dependent suppression of the parasite growth in the percentage of parasites (Fig. 1A). Among the four tested plant extracts, *T. africanum* crude extract showed the highest level of parasite growth inhibition at all parasite stages (Fig. 1A). Morphological alterations, such as cell shrinkage, and parasite fragmentation were observed following treatment with plant extract concentration of 50 μg/ml as well as after treatment with the positive control drug, chloroquine, in comparison with untreated parasites (Fig. 1B).

### Cytotoxicity of plant extracts

The cytotoxic potential of all included plant extracts at concentrations ranging from 1000 to 7.8 μg/ml (two-fold serial dilutions) were determined, and the mean IC_50_ values against HFF cells were calculated (Table 2). The methanolic and aqueous extracts from *C. procera*, the ethanolic extract from *P. undulata*, both the methanolic and ethanolic extracts from *C. colocynthis*, the methanolic extract from *P. undulata*, and the ethanolic extract from *O. basilicum* showed the highest cytotoxicity against HFF cells with mean IC_50_s of 2.9, 41.5, 55.5, 65.6, 88.0, 197.5, and 252.6 μg/ml, respectively. Extracts from plants *A. judaica, C. droserifolia, A. javanica, T. africanum, F. tenacissima,* and *V. tortilis* showed moderate-to-weak toxicity against HFF cells with mean IC_50_s of 316.8, 370.9, 378.1, 413.0, 519.0, and 554.5 μg/ml, respectively. Lastly, Extracts from *A. setifera* and *O. baccatus* were nontoxic or safe for HFF cells as cytotoxicity was not observed and their mean IC_50_s were > 1000 μg/ml of 1263.6 μg/ml, and 1179.0 μg/ml, respectively (Table 2).

### In vivo antimalarial activity of plant extracts

**Fig. 2:**
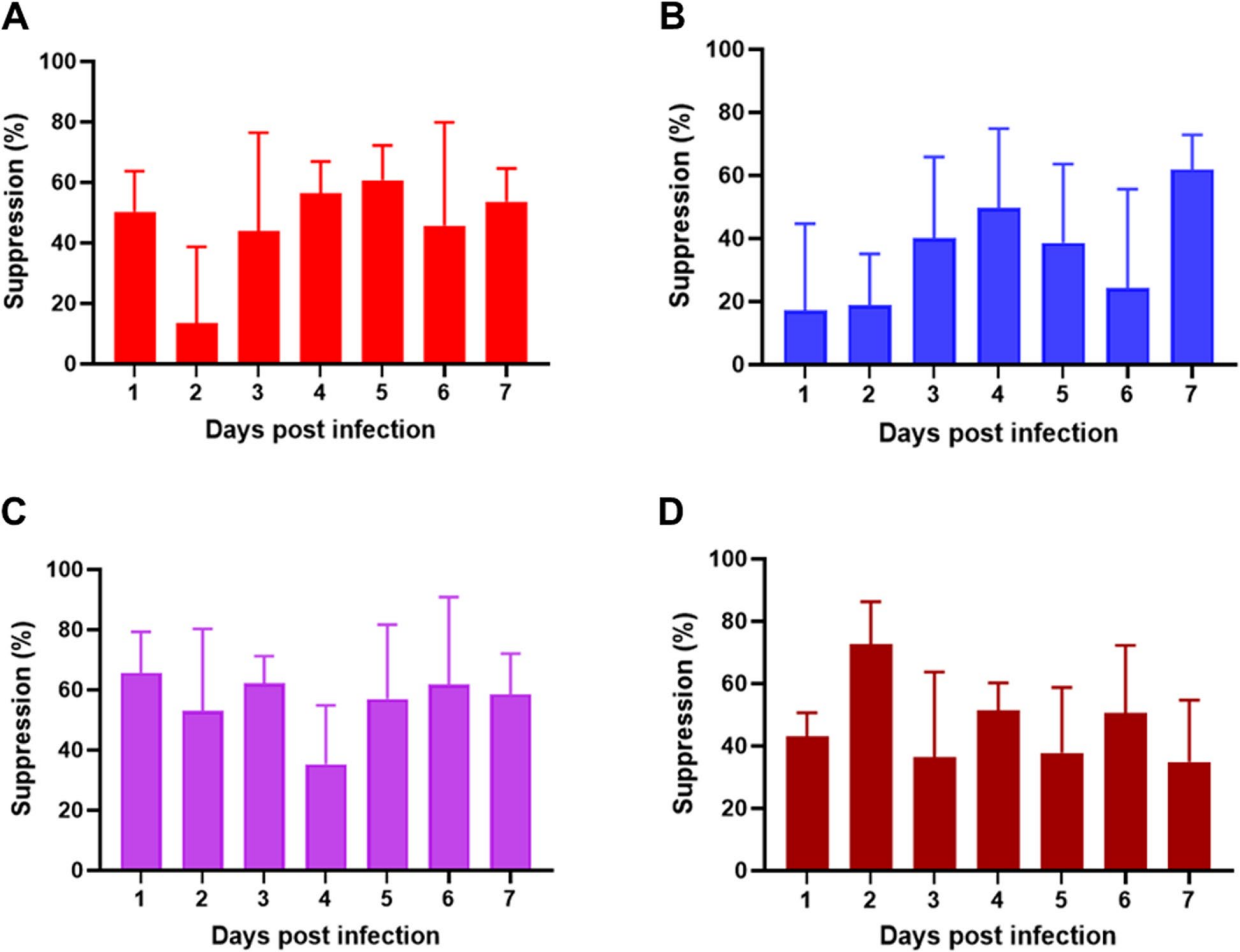
Suppression percentage of *Plasmodium yoelii* in mice induced by plant extracts at 1-week post-infection. Five mice were used per group. *Artemisia judaica* and *Cleome droserifolia* plant extracts were tested in one independent experiment that shared the same control, and *Trichodesma africanum* and *Vachellia tortilis* plant extracts were tested in another independent experiment that shared the same control. All mice were challenged by an intraperitoneal injection of approximately 1 × 10^7^*P. yoelii*-infected erythrocytes and then were treated orally with 100 mg/kg/day of each plant extract for 1 week. The mean and standard deviation of the parasite growth inhibition percentages were calculated against the untreated group mice from 24 h after challenge (dpi = 1) until 7 days post-infection (24 h after end course of treatment) (**A–D**) Parasite growth inhibition percentages in mice treated with *A. judaica* (**A**), *C. droserifolia* (**B**), *T. africanum* (**C**), or *V. tortilis* (**D**) plant extract. N.D. not detected

Their in-vitro results suggested that plant extracts from *T. africanum, C. droserifolia, A. judaica*, and *V. tortilis* have low or no cytotoxicity and might have activity against *Plasmodium* parasites. Therefore, we decided to evaluate their efficacy against *P. yoelii* in a murine malaria model. A chemotherapeutic test of each plant extract was performed beginning at the treatment start time of each extract (3 h post-challenge; day 0) and continuing through 6 days post-infection (the end of the course of the treatment). The four tested extracts each showed a time-dependent suppression of parasitemia through 7 days post-infection (Fig. 2). The mean level of parasite growth suppression observed after treatment with extracts of *A. judaica*, *C. droserifolia*, *T. africanum*, or *V. tortilis* ranged from 13.5–60.6%, 17.1–61.9, 35.2–65.5%, and 36.3–72.5%, respectively (Fig. 2, Table S2).

**Fig. 3:**
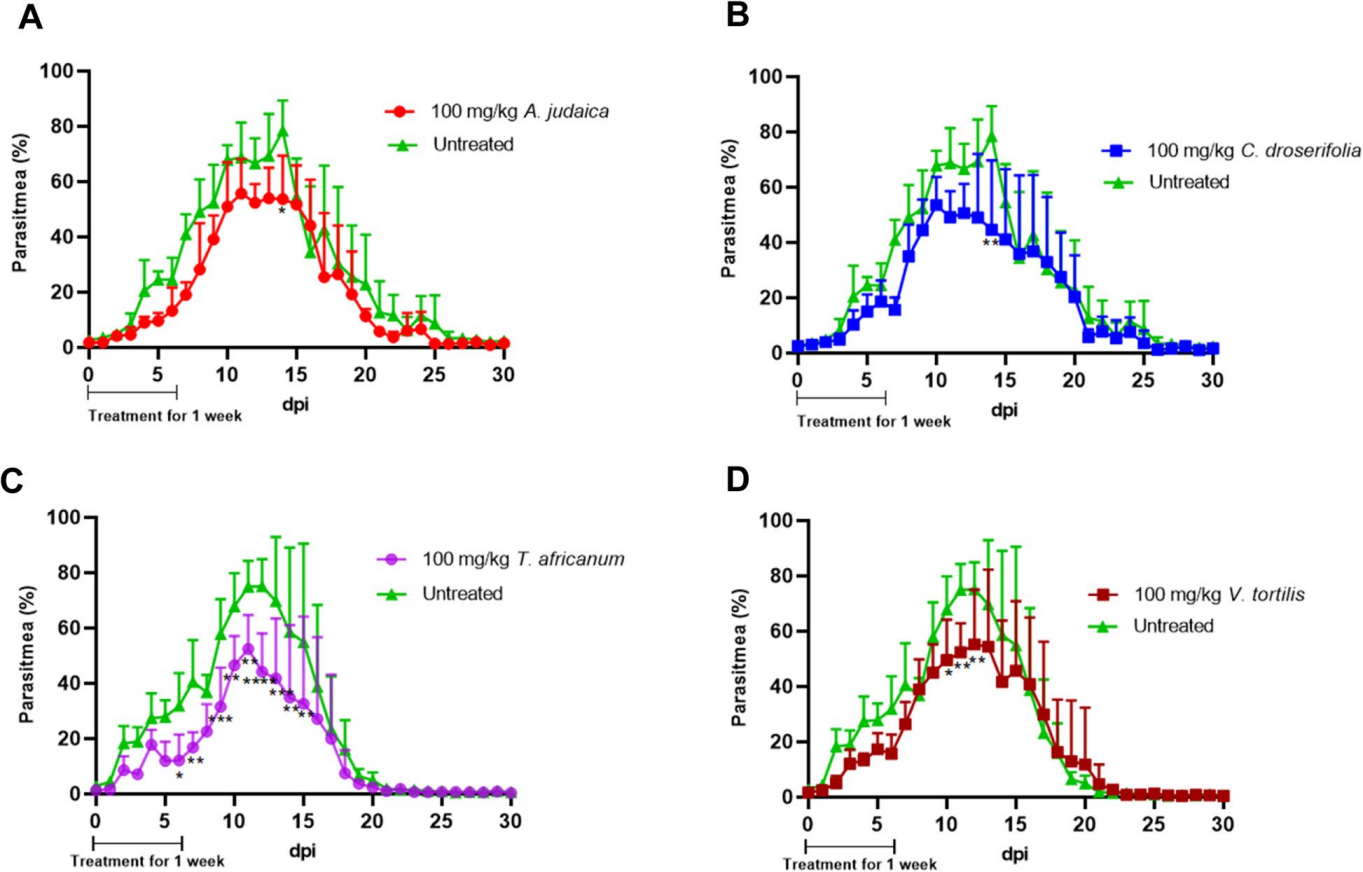
Effect of wild plant extracts on *Plasmodium yoelii* growth in mice through 30 days post-infection. Five mice were used in each group. *Artemisia judaica* and *Cleome droserifolia* plant extracts were tested in one independent experiment that shared the same control, and *Trichodesma africanum* and *Vachellia tortilis* plant extracts were tested in another independent experiment that shared the same control. Mean parasitemia % was monitored daily from day 0 (challenge day) until 30 days post-infection. All mice were challenged by an intraperitoneal injection of approximately 1 × 10^7^*Plasmodium yoelii-*infected erythrocytes and then treated orally with 100 mg/kg/day of plant extract for 1 week. The untreated group received only PBS. **A–D** The mean parasitemia % of *P. yoelii-*infected mice treated with *A. judaica* (**A**), *C. droserifolia* (**B**), *T. africanum* (**C**), or *V. tortilis* (**D**) extract. Data were analyzed by a two-way ANOVA followed by a Bonferroni test against the untreated group (*p < 0.05)

In the murine malaria model, a significantly reduced level of parasitemia was observed from 6 to 15 days post-infection after oral treatment with *T. africanum* extract (Fig. 3C), whereas there was no significant difference in the hematocrit, bodyweight change, or survival rate of *T. africanum* extract-treated mice as compared with mice in the untreated group (Figs. S3C, S4C, and S5C). Parasite growth suppression by *C. droserifolia* extract was not observed, except at day 14 post-infection (Fig. 3B). The hematocrit, bodyweight change, and survival rate of *C. droserifolia* extract-treated mice did not show any significant difference compared with untreated animals (Figs. S3B, S4B, and S5B). Although some parasite suppression efficacy was observed for *A. judaica* extract following treatment initiation through 6 days post-infection (Table S2; Fig. 2A), the level of parasite suppression at the peak of parasitemia was not significant, and *A. judaica* extract-treated mice took a similar length of time to recover as compared with the untreated mice (Fig. 3A); furthermore, the hematocrit, bodyweight percentage, and survival rate of these mice were not different from those of the untreated animals (Figs. S3A, S4A, and S5A). The in vivo parasite suppression induced by *V. tortilis* extract was only partial, but it was significant during the peak of parasitemia at days 10, 11, and 12 post-infection (Table S2, Fig. 3D); however, the hematocrit, bodyweight change, and survival rate of *V. tortilis* extract-treated mice were not significantly different from those of the untreated mice (Figs. S3D, S4D, and S5D).

## Discussion

*Trichodesma africanum* has been reported to have multiple medicinal uses (Table S1). The antibacterial efficacy of an oil extract of *T. africanum* was evaluated against the growth of three bacterial strains obtained from the American type culture collection (ATCC) (*Staphylococcus aureus* [ATCC 25923], *Escherichia coli* [ATCC 25922], and *Pseudomonas aeruginosa* [ATCC 27853]) as well as against the growth of methicillin-resistant *S. aureus* (MRSA) isolates, and its antifungal activity was tested against *Candida albicans* [37]. The chemical constituents of *T. africanum* include essential oils, steroids, coumarins, flavonoids, phenolics, alkaloids, and glycosides [38]. Its chemical constituents might be involved in its biological activity, as flavonoids had been reported to have antiprotozoal activity, specifically anti-leishmanial and anti-trypanosomal activities [39].

*Trichodesma africanum* collected from Saudi Arabia was reported to have weak antimalarial efficacy in vitro against a chloroquine-sensitive strain of *P. falciparum* with an IC_50_ value of 32.0 μg/ml (SI of 2) [40]. Here, *T. africanum* from an Egyptian desert was found to possess a moderate-to-weak activity in vitro against *P. falciparum* with a mean IC_50_ value of 11.7 μg/ml (SI of 35.2; Table 2). These results suggest that the efficacy of plant extracts can vary owing to differences in the area, time of day, and season of plant collection, extraction procedures used in collection, and cell lines used for the determination of its cytotoxic potential. Although the antimalarial activity of *T. africanum* was previously examined in vitro, our study is the first to illustrate the antimalarial efficacy of a *T. africanum* extract in a mouse model of malaria.

*Cleome droserifolia*, which is facing extinction, is found in tropical and subtropical areas, such as North Africa and India [24, 41, 42]. *C. droserifolia* is an important plant species owing to its historical use in traditional medicine in Egypt [43–45]. Regarding its medicinal uses, it has an immediate effect on abdominal and rheumatic pain, is anti-inflammatory, and is also effective for improving wound healing and treating snake bites and scorpion stings [43–46]. These effects are attributed to the rubefacient, antimicrobial, analgesic, antipyretic, antioxidant, and anti-inflammatory activities of its components [45–49], which include flavonoids, glycosides, carbohydrates, buchariol, teucladiol, daucosterol, cardenolides, saponins, sterols, tannins, catechins, triterpenes and sesquiterpenes as well as a newly described alkaloid found in its aerial parts [50–53]. *C. droserifolia* was previously reported to have an antibacterial effect [54], but little is known about its antiprotozoal efficacy. Although there is no literature on the effect of *C. droserifolia* extract on malaria, another species in the *Cleome* genus (e.g., *Cleome rutidosperma*) was reported to have moderate anti-plasmodial activity against *P. falciparum* CQS D10 strain in vitro (IC_50_ value: 34.4 μg/ml) [55]. In the present study, *C. droserifolia* was evaluated with both in vitro and in vivo assays. It had a moderate-to-weak inhibitory effect, with an IC_50_ value of 32.1 μg/ml and an SI of 12.9 in vitro against *P. falciparum*, and partially inhibited murine malaria in a short-term treatment of 100 mg/kg/day.

Recently, *A. tortilis* plant name was changed and become well known as *V. tortilis* according to [28], therefore, all information about *V. tortilis* in this discussion section is reported under its old name, *A. tortilis*. This plant is one of 1200 species of *Acacia*. It grows in tropical and subtropical areas with temperatures in the range of 40–45 °C in summer and < 5 °C in winter, such as locations in African, Australian, and Arabian countries [56]. *A. tortilis* possesses multiple medicinal and pharmacological properties, e.g., antidiabetic, antifungal, antidiarrheal, antitussive, and anti-inflammatory [57], but little is known about its antiprotozoal potential. Despite of the potent effectiveness of the methanolic extract of *A. tortilis* collected from Kenya as an anti-parasitic treatment, there are conflicting reports regarding its antimalarial activity. Some studies found that it had moderate efficacy against *P. falciparum* [57, 58]. In contrast, although the first in a pair of studies initially showed that *A. tortilis* root bark showed antimalarial activity, its follow-up study did not consider it to be an antimalarial candidate [57, 59]. In the present work, the methanolic extract from *A. tortilis* seeds collected from an Egyptian desert showed moderate-to-weak activity in vitro against *P. falciparum* (3D7). These results suggest that the efficacy of *V. tortilis* extract against malaria may be correlated to its medicinal uses (i.e., as an antimicrobial or other treatment) or to its chemical constituents and may vary depending on the extracted plant part and area from which the plant was collected. In vivo parasite suppression against *P. yoelii* in mice was observed until 6 days post-infection, and this extract significantly suppressed the parasitemia during its peak from days 10–12 post-infection, suggesting that it has partial efficacy against murine malaria.

*Artemisia judaica* belongs to the family *Asteraceae*, which is one of the largest families of angiosperms and contains 1600 to 1700 genera and about 24,000 species distributed worldwide [60]. Known as shih in the Middle-East, *A. judaica* is an aromatic shrub found mainly in the deserts of the Middle-East, Egypt, and serval North African countries and is traditionally used as an anthelmintic drug [61]. Although *A. judaica* has not been previously reported to have antimalarial efficacy, numerous other *Artemisia* species have been found to have antimalarial activity, including *Artemisia nilagirica* [62], *Artemisia maciverae* (chloroform extract) [63], *Artemisia maritima* (ethanolic and petroleum extracts), *Artemisia nilegarica*, *Artemisia japonica* [64], *Artemisia ciniformis*, *Artemisia biennis*, and *Artemisia turanica* [65]. Artemisinin, the well-known conventional drug discovered by Chinese scientists obtained from *Artemisia annua* [66], has also been found in several other species of *Artemisia*, including *Artemisia lancea*, *Artemisia apiacea* [67], *Artemisia vulgaris* [68], *A. japonica* [69], *Artemisia sieberi* [70], *Artemisia absinthium* [71], *Artemisia dubia*, and *Artemisia indica* [72]. There is no available information about the presence of artemisinin in *A. judaica*.

*Plasmodium* often develops drug resistance, and there is now evidence of resistance to artemisinin drugs [73]. Therefore, there is an urgent need to find novel candidates for the development of drugs to treat *Plasmodium*; other *Artemisia* plants, such as *A. judaica*, and other plant species may be useful sources. Several *Artemisia* species have been evaluated for their antimalarial activity in rodent malaria models. *A. vulgaris* showed potent activity without toxicity when administrated orally to mice infected with *P. yoelii*, according to the results of a 4-day suppressive test performed following treatment with high doses (500 mg/kg and 1000 mg/kg) of *A. vulgaris* extract [74]. Furthermore, the efficacy of an ethanolic extract of *A. vulgaris* leaves was confirmed against *Plasmodium berghei* ANKA strain in ICR mice. Treatment with doses of 500, 750, and 1000 mg/kg of this extract significantly reduced parasitemia by 79.3, 79.6, and 87.3%, respectively [75]. *A. sieberi* from Iran showed antimalarial efficacy against *P. berghei* in NMRI mice, reducing some pathophysiological signs of malaria [76]. An infusion of *A. annua* (tea) failed to cause any reduction to the parasitemia caused by *Plasmodium chabaudi* in OF1 mice [77], whereas the oral administration of dried whole *A. annua* leaves killed these parasites more effectively than did a comparable dose of the pure drug artemisinin in C57BL/6 mice [78].

Here, a methanolic leaf extract of *A. judaica* was found to possess moderate-to-weak antimalarial activity against *P. falciparum* with no apparent cytotoxicity along with a moderate efficacy against murine malaria at a lower dose (100 mg/kg/day) than used in previous studies. However, it did not cause a significant reduction in the parasitemia of *P. yoelii* during the peak of infection in mice.

*Calotropis procera* has been reported to have multiple biological and medicinal uses [79]. An ethanolic extract of this plant was reported to have a schizonticidal effect in vitro [80]. Furthermore, fractions from the leaf extract show anti-plasmodial activity [81]. However, in our study, neither the methanolic nor the aqueous extract of *C. procera* flowers showed any efficacy in vitro against *P. falciparum* (3D7) when administered at a dose of 100 μg/ml.

Three rodent-specific *Plasmodium* species, *P. berghei*, *P. yoelii*, and *P. chabaudi*, are commonly used in animal models of malaria; these models exhibit different manifestations of the human disease. In vitro cultures of these parasites are not well established; thus, they require maintenance in mice [82]. Here, we used a *P. yoelii* mouse model to evaluate the antimalarial efficacy of four plant extracts. In previous plant extract treatment trials of *P. yoelii* in Swiss albino mice, treatment with aqueous or ethanolic extracts of *Phyllanthus amarus* at doses of 200, 400, 800, and 1600 mg/kg/day was performed until 6 days post-infection; respectively, the aqueous extract induced 56.0, 68.0, 77.9, and 81.2% parasite suppression, and the ethanolic extract induced 51.7, 67.9, 74.2, and 52.3% parasite suppression [83]. Another study used 1.25 g/kg of methanolic extract from *Nigella sativa* seeds. Parasite suppression of 84.6, 89.2, and 94% was observed at days 6, 7, and 8 post-infection with *P. yoelii* nigeriensis, respectively [84]. Furthermore, the efficacies of methanolic-chloroform (MC) and methanolic-aqueous (MA) extracts from *Brucei mollis* collected from India were evaluated against *P. yoelii* N-67 (chloroquine-resistant strain [CQR]); they had respective median effective doses 50 (ED_50_s) of 30 mg/kg/day and 72 mg/kg/day at 4 days post-infection and of 66 mg/kg/day and 79 mg/kg/day at 6 days post-infection [85]. In the present study, treatment with 100 mg/kg/day methanolic extracts of *A. judaica, C. droserifolia, T. africanum*, or *V. tortilis* from 0 to 6 days post-infection each caused significant parasite suppression, with mean suppression percentages ranges of 13.5–60.6%, 17.1–61.9, 35.2–65.5%, and 36.3–72.5%, respectively, despite the extract dose being lower compared with previously reported studies (Table S2).

## Conclusions

This study showed that crude extracts of four wild plants collected from Egypt had antimalarial efficacy against the human malaria-causing parasite *P. falciparum* in vitro and against the murine malaria-causing parasite *P. yoelii* in a mouse model. Although the administration of these extracts at a dose of 100 mg/kg/day for a 7-day course of treatment did not achieve 100% inhibition of *P. yoelii* growth in BALB/c mice, the parasite suppression data suggests that these extracts may have potent antimalarial activity. Their efficacies are likely correlated with their multiple medicinal uses and their chemical constituents. Among the four tested candidates, the *T. africanum* crude extract possessed the highest parasite suppression ability in a short-term treatment course in vivo and had the highest IC_50_ in vitro against the human malaria-causing parasite *P. falciparum*, whereas *V. tortilis* extract showed moderate-to-weak effect against *P. falciparum* in vitro and induced partial inhibition against *P. yoelii* in vivo. These data support the use of these extracts in the future development of an antimalarial therapeutic. Further study will be needed to understand the mechanism of action and identify the main biological components of these crude extracts.

## Supplementary Information

**Additional file 1: Table S1.** The plants used in this study and their reported medicinal uses. **Table S2.** Chemotherapeutic test of four plant extracts against the growth of *Plasmodium yoelii* in mice. **Figure S1.** Sampling map of the plant samples collected in Egypt. **Figure S2.** Images of the collected plant materials. **Figure S3.** Effect of wild plant extracts on the growth of *plasmodium yoelii* in male BALB/c mice. **Figure S4.** Effect of wild plant extracts on bodyweight change in *Plasmodium*-infected mice. **Figure S5.** Effect of wild plant extracts on survival rate of *Plasmodium*-infected mice.

## Data Availability

All data generated or analysed during this study are included in this published manuscript and its supplementary information file.

## Abbreviations

P. falciparum: Plasmodium falciparum
*P. yoelii*: *Plasmodium yoelii*
i.p: intraperitoneal
IC_50_: Half maximal inhibitory concentration 50
dpi: days post infection
HFF: Human foreskin fibroblast
ATCC: American type culture collection
SI: Selectivity index
MRSA: Methicillin-resistant *staphylococcus aureus*
M80%: Methanol 80%
E70: Ethanol 70%
aq.: aqueous
PBS: Phosphate-buffered saline
pRBC: parasitized-RBCs
CQR: Chloroquine resistant
MC: Methanolic-chloroform
MA: Methanolic-aqueous
ED_50_: median effective dose 50

## References

[1] World Health Organization (WHO) (2020). World Malaria Report 2020 Medicines for Malaria Venture.

[2] White NJ, Pukrittayakamee S, Hien TT, Faiz MA, Mokuolu OA, Dondorp AM (2014). Malaria. Lancet.

[3] Garrido-Cardenas JA, González-Cerón L, Manzano-Agugliaro F, Mesa-Valle C (2019). *Plasmodium* genomics: an approach for learning about and ending human malaria. Parasitol Res.

[4] Trampuz A, Jereb M, Muzlovic I, Prabhu RM (2003). Clinical review: severe malaria. Crit Care.

[5] Kremsner PG, Krishna S (2004). Antimalarial combinations. Lancet.

[6] Payne D (1987). Spread of chloroquine resistance in *plasmodium falciparum*. Parasitol Today.

[7] Taylor WR, White NJ (2004). Antimalarial drug toxicity. Drug Saf.

[8] Dias DA, Urban S, Roessner U (2012). A historical overview of natural products in drug discovery. Metabolites.

[9] Willcox M, Benoit-Vical F, Fowler D, Bourdy G, Burford G, Giani S, Graziose R, Houghton P, Randrianarivelojosia M, Rasoanaivo P (2011). Do ethnobotanical and laboratory data predict clinical safety and efficacy of anti-malarial plants?. Malar J.

[10] Rasoanaivo P, Wright CW, Willcox ML, Gilbert B (2011). Whole plant extracts versus single compounds for the treatment of malaria: synergy and positive interactions. Malar J.

[11] Wink M (2012). Medicinal plants: a source of anti-parasitic secondary metabolites. Molecules..

[12] World Health Organization (WHO). 2021. Who-recommends-groundbreaking-malaria-vaccine-for-children-at-risk. https://www.who.int/news/item/06-10-2021. Accessed 24 Feb 2022.

[13] Mostafa N, Singab A (2018). Prospective of herbal medicine in Egypt. Med Chem (Los Angeles).

[14] Hout S, Chea A, Bun SS, Elias R, Gasquet M, Timon-David P, Balansard G, Azas N (2006). Screening of selected indigenous plants of Cambodia for antiplasmodial activity. J Ethnopharmacol.

[15] Bagavan A, Rahuman AA, Kaushik NK, Sahal D (2011). In vitro antimalarial activity of medicinal plant extracts against *plasmodium falciparum*. Parasitol Res.

[16] Rufin Marie TK, Mbetyoumoun Mfouapon H, Madiesse Kemgne EA, Jiatsa Mbouna CD, Tsouh Fokou PV, Sahal D, Fekam Boyom F (2018). Anti-*plasmodium falciparum* activity of extracts from 10 Cameroonian medicinal plants. Medicines.

[17] Kwansa-Bentum B, Agyeman K, Larbi-Akor J, Anyigba C, Appiah-Opong R. In Vitro Assessment of Antiplasmodial Activity and Cytotoxicity of Polyalthia longifolia Leaf Extracts on Plasmodium falciparum Strain NF54, Malaria Research and Treatment. 2019;2019:9. Article ID 6976298. 10.1155/2019/6976298. 10.1155/2019/6976298 PMC6360585 30805129

[18] Shittu I, Emmanuel A, Nok AJ. Antimalaria Effect of the Ethanolic Stem Bark Extracts of Ficus platyphylla Del. J Parasitology Res. 2011;2011:5. Article ID 618209. 10.1155/2011/618209 10.1155/2011/618209 PMC3228363 22174991

[19] Tepongning RN, Yerbanga SR, Dori GU, Lucantoni L, Lupidi G, Habluetzel A (2013). In vivo efficacy and toxicity studies on *Erythrina senegalensis* and *Khaya ivorensis* used as herbal remedies for malaria prevention in Cameroon. Eur J Med Plants.

[20] Chandel S, Bagai U, Vashishat N (2012). Antiplasmodial activity of *Xanthium strumarium* against *plasmodium berghei*-infected BALB/c mice. Parasitol Res.

[21] Chutoam P, Klongthalay S, Somsak V (2015). Effect of crude leaf extract of *Bauhinia strychnifolia* in BALB/c mice infected with *plasmodium berghei*. Malar Cont Elimination.

[22] Mohd Ridzuan MAR, Sow A, Noor Rain A, Mohd Ilham A, Zakiah I (2007). Eurycoma longifolia extract-artemisinin combination: parasitemia suppression of *plasmodium yoelii*-infected mice. Trop Biomed.

[23] Ishih A, Miyase T, Ohori K, Terada M (2003). Different responses of three rodent *plasmodia* species, *plasmodium yoelii* 17XL, *P. berghei* NK65 and *P. chabaudi* AS on treatment with febrifugine and isofebrifugine mixture from *Hydrangea macrophylla* var. Otaksa leaf in ICR mice. Phytother Res.

[24] Boulos L (1999). Flora of Egypt, Volume 1: Azollaceae - Oxalidaceae.

[25] Boulos L (1999). Flora of Egypt, volume 2: Geraniaceae–Boraginaceae.

[26] Boulos L (2002). Flora of Egypt, volume 3: Verbenaceae-Compositae.

[27] Boulos L (2009). Flora of Egypt checklist - revised Annotated Edition.

[28] POWO. Plants of the world online. Facilitated by the Royal Botanic Gardens, Kew. 2019. Available from: http://www.plantsoftheworldonline.org/. Accessed June 2021.

[29] Leesombun A, Boonmasawai S, Nishikawa Y (2019). Ethanol extracts from Thai plants have anti-*plasmodium* and anti-*toxoplasma* activities in vitro. Acta Parasitol.

[30] Pagmadulam B, Tserendulam D, Rentsenkhand T, Igarashi M, Sawa R, Nihei CI, Nishikawa Y (2020). Isolation and characterization of antiprotozoal compound-producing Streptomyces species from Mongolian soils. Parasitol Int.

[31] Leesombun A, Iijima M, Pagmadulam B, Orkhon B, Doi H, Issiki K, Sawa R, Nihei CI, Nishikawa Y (2021). Metacytofilin has potent anti-malarial activity. Parasitol Int.

[32] Johnson JD, Dennull RA, Gerena L, Lopez-Sanchez M, Roncal NE, Waters NC (2007). Assessment and continued validation of the malaria SYBR green I-based fluorescence assay for use in malaria drug screening. Antimicrob Agents Chemother.

[33] Ariefta NR, Koseki T, Nishikawa Y, Shiono Y (2021). Spirocollequins a and B, new alkaloids featuring a spirocyclic isoindolinone core, from Colletotrichum boninense AM-12-2. Tetrahedron Lett.

[34] Rasoanaivo P, Deharo E, Ratsimanga-Urverg S, Frappier F, Willcox M, Rasoanaivo P, Bodeker G (2004). Guidelines for the Non-clinical Evaluation of the Efficacy of Traditional Antimalarials. Traditional medicine plants and malaria.

[35] Kweyamba PA, Zofou D, Efange N, Assob JC, Kitau J, Nyindo M (2019). In vitro and in vivo studies on anti-malarial activity of *Commiphora africana* and *Dichrostachys cinerea* used by the Maasai in Arusha region, Tanzania. Malar J.

[36] Peters W (1975). The four-day suppressive in vivo antimalarial test. Ann Trop Med Parasitol.

[37] Jaradat NA, Zaid AN, Abuzant A, Shawahna R (2016). Investigation the efficiency of various methods of volatile oil extraction from *Trichodesma africanum* and their impact on the antioxidant and antimicrobial activities. J Intercult Ethnopharmacol.

[38] El-Moaty, A. Active Constituents and antimicrobial activity of Trichodesma africanum (L.) R. Br. var. heterotrichum Bornm. & Kneuck. Egyptian J Agricultural Sciences. 2009;60(4):357-65.

[39] Tasdemir D, Kaiser M, Brun R, Yardley V, Schmidt TJ, Tosun F, Rüedi P (2006). Antitrypanosomal and antileishmanial activities of flavonoids and their analogues: in vitro, in vivo, structure-activity relationship, and quantitative structure-activity relationship studies. Antimicrob Agents Chemother.

[40] Abdel-Sattar E, Maes L, Salama MM (2010). In vitro activities of plant extracts from Saudi Arabia against malaria, leishmaniasis, sleeping sickness and Chagas disease. Phytother Res.

[41] Batanouny KH, Aboutabl E, Shabana M, Soliman F (1999). Wild medicinal plants in Egypt.

[42] Kamel WM, Abd El-Ghani MM, El-Bous M (2010). Cleomaceae as a distinct family in the flora of Egypt. Afr J Plant Sci Biotechnol.

[43] Aparadh VT, Mahamuni RJ, Karadge BA (2012). Taxonomy and physiological studies in spider flower (*Cleome* species): a critical review. Plant Sci Feed.

[44] Rahman MA, Mossa JS, Al-Said MS, Al-Yahya MA (2004). Medicinal plant diversity in the flora of Saudi Arabia 1: a report on seven plant families. Fitoterapia..

[45] Moustafa A, Sarah R, Qiqa S, Mansour S, Alotaibi M (2019). *Cleome droserifolia*: An Egyptian natural heritage facing extinction. Asian J Plant Sci Res.

[46] Sarhan WA, Azzazy HM, El-Sherbiny IM (2016). Honey/chitosan nanofiber wound dressing enriched with *Allium sativum* and *Cleome droserifolia*: enhanced antimicrobial and wound healing activity. ACS Appl Mater Interfaces.

[47] El-Ghazali GE, Al-Khalifa KS, Saleem GA, Abdallah EM (2010). Traditional medicinal plants indigenous to Al-Rass province, Saudi Arabia. J Med Plant Res.

[48] Abd El-Gawad AM, El-Amier YA, Bonanomi G (2018). Essential oil composition, antioxidant and allelopathic activities of *Cleome droserifolia* (Forssk). Delile Chem Biodiversity.

[49] Panicker NG, Balhamar SO, Akhlaq S, Qureshi MM, Rehman NU, Al-Harrasi A, Hussain J, Mustafa F (2020). Organic extracts from *Cleome droserifolia* exhibit effective caspase-dependent anticancer activity. BMC Complement Med Ther.

[50] Aboushoer MI, Fathy HM, Abdel-Kader MS, Goetz G, Omar AA (2010). Terpenes and flavonoids from an Egyptian collection of *Cleome droserifolia*. Nat Prod Res.

[51] Hussain J, Khan H, Ali L, Latif Khan A, Ur Rehman N, Jahangir S, Al-Harrasi A (2015). A new indole alkaloid from *cleome droserifolia*. Helv Chim Acta.

[52] Abdullah W, Elsayed WM, Abdelshafeek KA, Nazif NM, Singab AN (2016). Chemical constituents and biological activities of *Cleome* genus: a brief review. Int J Pharmacogn Phytochem Res.

[53] Singh H, Mishra A, Mishra AK (2018). The chemistry and pharmacology of *Cleome* genus: a review. Biomed..

[54] Muhaidat R, Al-Qudah MA, Samir O, Jacob JH, Hussein E, Al-Tarawneh IN, Bsoul E, Orabi ST (2015). Phytochemical investigation and in vitro antibacterial activity of essential oils from *Cleome droserifolia* (Forssk.) Delile and C. trinervia Fresen. (Cleomaceae). S Afr J Bot.

[55] Bose A, Smith PJ, Lategan CA, Gupta JK, Si S (2010). Studies on in vitro antiplasmodial activity of *Cleome rutidosperma*. Acta Pol Pharm Drug Res.

[56] Ibrahim AA, Aref IM (2000). Host status of thirteen *Acacia* species to *Meloidogyne javanica*. J Nematol.

[57] Yadav P, Kant R, Kothiyal P (2013). A review on *Acacia tortilis*. Int J Pharm Phytopharm Res.

[58] Kigondu EV, Rukunga GM, Keriko JM, Tonui WK, Gathirwa JW, Kirira PG, Irungu B, Ingonga JM, Ndiege IO (2009). Anti-parasitic activity and cytotoxicity of selected medicinal plants from Kenya. J Ethnopharmacol.

[59] Nguta JM, Mbaria JM (2013). Brine shrimp toxicity and antimalarial activity of some plants traditionally used in treatment of malaria in Msambweni district of Kenya. J Ethnopharmacol.

[60] Hussain A, Hayat MQ, Sahreen S, Ain QU, Bokhari SA (2017). Pharmacological promises of genus *Artemisia* (Asteraceae): a review. Proc Pakistan Acad Sci: B Life Environ Sci.

[61] Wyk BEV, Wink M (2004). Medicinal plants of the world: An illustrated scientific guide to important medicinal plants and their uses.

[62] Panda S, Rout JR, Pati P, Ranjit M, Sahoo SL (2018). Antimalarial activity of *Artemisia nilagirica* against *Plasmodium falciparum*. J Parasit Dis.

[63] Ene AC, Atawodi SE, Ameh DA, Ndukwe GI, Kwanashie HO (2009). Bioassay-guided fractionation and in vivo antiplasmodial effect of fractions of chloroform extract of *Artemisia maciverae* Linn. Acta Trop.

[64] Valecha NE, Biswas S, Badoni V, Bhandari KS, Sati OP (1994). Antimalarial activity of *Artemisia japonica*, *Artemisia maritima* and *Artemisia nilegarica*. Indian J Pharm.

[65] Mojarrab M, Naderi R, Afshar FH (2015). Screening of different extracts from *Artemisia* species for their potential antimalarial activity. Iran J Pharm Sci.

[66] Covello PS (2008). Making artemisinin. Phytochemistry..

[67] Qian GP, Yang YW, Ren QL (2005). Determination of artemisinin in *Artemisia annua* L. by reversed phase HPLC. J Liq Chromatogr Relat Technol.

[68] Numonov S, Sharopov F, Salimov A, Sukhrobov P, Atolikshoeva S, Safarzoda R, Habasi M, Aisa HA (2019). Assessment of artemisinin contents in selected *Artemisia* species from Tajikistan (Central Asia). Medicines.

[69] Rashmi TR, Francis MS, Murali S (2014). Determination of Artemisinin in selected *Artemisia* L. species by HPLC. Indo Am J Pharm.

[70] Arab HA, Rahbari S, Rassouli A, Moslemi MH, Khosravirad F (2006). Determination of artemisinin in *Artemisia sieberi* and anticoccidial effects of the plant extract in broiler chickens. Trop Anim Health Prod.

[71] Zia M, Mannan A, Chaudhary MF (2007). Effect of growth regulators and amino acids on artemisinin production in the callus of *Artemisia absinthium*. Pak J Bot (Pakistan).

[72] Mannan A, Shaheen N, Arshad W, Qureshi RA, Zia M, Mirza B. Hairy roots induction and artemisinin analysis in Artemisia dubia and Artemisia indica. Afr J Biotechnol. 2008;7(18):3288-92. 17 September, 2008. Available online at http://www.academicjournals.org/AJB. ISSN 1684–5315 © 2008 Academic Journals.

[73] Dondorp AM, Nosten F, Yi P, Das D, Phyo AP, Tarning J, Lwin KM, Ariey F, Hanpithakpong W, Lee SJ, Ringwald P, Silamut K, Imwong M, Chotivanich K, Lim P, Herdman T, An SS, Yeung S, Singhasivanon P, Day NP, Lindegardh N, Socheat D, White NJ (2009). Artemisinin Resistance in *Plasmodium falciparum* Malaria. N Engl J Med.

[74] Kodippili K, Daya Ratnasooriya W, Premakumara S, Udagama PV. An investigation of the antimalarial activity of Artemisia vulgaris leaf extract in a rodent malaria model. Int J Green Pharm. 2011;5(4). https://hdl.handle.net/70130/5248/. P-ISSN0973-8258 E-ISSN - 1998-4103.

[75] Bamunuarachchi GS, Ratnasooriya WD, Premakumara S, Udagama PV (2013). Antimalarial properties of *Artemisia vulgaris* L. ethanolic leaf extract in a *plasmodium berghei* murine malaria model. J Vector Borne Dis.

[76] Nahrevanian H, Sheykhkanlooye Milan B, Kazemi M, Hajhosseini R, Soleymani Mashhadi S, Nahrevanian S. Antimalarial effects of Iranian flora Artemisia sieberi on Plasmodium berghei in vivo in mice and phytochemistry analysis of its herbal extracts. Malaria research and treatment. Hindawi Publishing Corporation Malaria Research and Treatment. 2012;2012:8. 10.1155/2012/727032. Article ID 727032. 10.1155/2012/727032 PMC3270465 22315701

[77] Atemnkeng MA, Chimanuka B, Dejaegher B, Vander Heyden Y, Plaizier-Vercammen J (2009). Evaluation of *Artemisia annua* infusion efficacy for the treatment of malaria in *plasmodium chabaudi chabaudi* infected mice. Exp Parasitol.

[78] Elfawal MA, Towler MJ, Reich NG, Golenbock D, Weathers PJ, Rich SM (2012). Dried whole plant *Artemisia annua* as an antimalarial therapy. PLoS One.

[79] Meena AK, Yadav AK, Niranjan US, Singh B, Nagariya AK, Sharma K, Gaurav A, Sharma S, Rao MM (2010). A review on *Calotropis procera* Linn and its ethnobotany, phytochemical, pharmacological profile. Drug Invent Today.

[80] Sharma P, Sharma JD (2000). In-vitro schizonticidal screening of *Calotropis procera*. Fitoterapia.

[81] Mudi SY, Bukar A. Anti-plasmodia activity of leaf extracts of Calotropis procera Linn. Biokemistri. 2011;23(1). Available online at https://www.bioline.org.br/bk.

[82] Huang BW, Pearman E, Kim CC. Mouse models of uncomplicated and fatal malaria. Bio Protoc. 2015;5(13):e1514. 10.21769/bioprotoc.1514. 10.21769/bioprotoc.1514 PMC4520541 26236758

[83] Ajala TO, Igwilo CI, Oreagba IA, Odeku OA (2011). The antiplasmodial effect of the extracts and formulated capsules of *Phyllanthus amarus* on *plasmodium yoelii* infection in mice. Asian Pac J Trop.

[84] Okeola VO, Adaramoye OA, Nneji CM, Falade CO, Farombi EO, Ademowo OG (2011). Antimalarial and antioxidant activities of methanolic extract of *Nigella sativa* seeds (black cumin) in mice infected with *plasmodium yoelli nigeriensis*. Parasitol..

[85] Prakash A, Sharma SK, Mohapatra PK, Bhattacharjee K, Gogoi K, Gogoi P, Mahanta J, Bhattacharyya DR (2013). In vitro and in vivo antiplasmodial activity of the root extracts of *Brucea mollis* wall. Ex Kurz. Parasitol Res.

