# Supplementary material for: Wild Egyptian medicinal plants show in vitro and in vivo cytotoxicity and antimalarial activities
Source: BMC Complement Med Ther. 2022 May 12;22:130. doi: 10.1186/s12906-022-03566-5 (PMC9101831; doi:10.1186/s12906-022-03566-5)
Supplement: Supplementary file 1 — Additional file 1: Table S1. The plants used in this study and their reported medicinal uses. Table S2. Chemotherapeutic test of four plant extracts against the growth of Plasmodium yoelii in mice. Figure S1. Sampling map of the plant samples collected in Egypt. Figure S2. Images of the collected plant materials. Figure S3. Effect of wild plant extracts on the growth of plasmodium yoelii in male BALB/c mice. Figure S4. Effect of wild plant extracts on bodyweight change in Plasmodium-infected mice. Figure S5. Effect of wild plant extracts on survival rate of Plasmodium-infected mice. [file 12906_2022_3566_MOESM1_ESM.doc]

**Supplemental information**

**Table S1. The plants used in this study** and their medicinal uses

| **Medicinal use** [References] | **Plant family** | **Coordinates** | | **Plant Extract** |
| --- | --- | --- | --- | --- |
|  |  | **Longitude**  **(E)** | **Latitude**  **(N)** |  |
| Antifungal, antimicrobial, nematocidal, used in diuretic, and urinary trouble [1] | Amaranthaceae | 33°16'19.6" | 26°29'11.5" | *Aerva javanica (Burm.f.) Juss. ex Schult.* |
| Anti-inflammatory [2] | Amaranthaceae | 33°14'42.4" | 26°28'44.1" | *Anabasis setifera Moq.* |
| Antihelmintic, expectorant, and analgesic [3] | Asteraceae | 33°14'09.6" | 26°28'31.7" | *Artemisia judaica L.* |
| Antirheumatic, antifungal, molluscicides, leprosy treatment, bronchial asthma and skin affection [4] ,anti-scorpion bite [5], Antimalarial [6–8] | Apocynaceae | 33°13'39.6" | 26°28'16.1" | *Calotropis procera (Aiton) Dryand.* |
| Anti-inflammatory, antioxidant, anti-hypoxic, anti-coagulant and anti-tumor, analgesic [9] | Asteraceae | 32°47'56.0" | 26°23'13.7" | *Carthamus tinctorius L.* |
| Antimicrobial, antimicrobial and anticancer [10] | Cucurbitaceae | 32°47'59.1" | 26°25'55.1" | *Citrullus colocynthis (L.) Schrad.* |
| For treatment for urinary tract pains, diabetes, wounds, dermatitis, and antimicrobial [3] | Cleomaceae | 32°46'26.3" | 26°17'54.5" | *Cleome droserifolia (Forssk.) Delile* |
| Hepatoprotective, antioxidant [11] antibacterial, antinociceptive, and antipyretic [12] | Urticaceae | 32°46'24.6" | 26°17'59.1" | *Forsskaolea tenacissima L.* |
| Diuretic, antiseptic, antitussive [13] | Resedaceae | 32°46'31.6" | 26°18'05.1" | *Ochradenus baccatus Delile* |
| Antimicrobial [14] | Lamiaceae | 32°46'42.9" | 26°18'03.0" | *Ocimum basilicum L.* |
| Antiulcerogenic [15], and anti-inflammatory [16] | Asteraceae | 32°46'31.8" | 26°18'44.5" | *Pulicaria undulata (L.) C.A.Mey.* |
| Antitumor [17] antimicrobial, and antifungal [18] | Boraginaceae | 32°46'47.9" | 26°19'40.2" | *Trichodesma africanum (L.) Sm.* |
| Antitussive [5], ocular infection treatment, and jaundice [3], Antifungal [19] | Fabaceae | 32°47'01.9" | 26°20'27.9" | *Vachellia tortilis subsp. raddiana (Savi) Kyal. & Boatwr.* |

The name, coordinates of the collection sites, family as well as the reported medicinal uses of each plant used in this study were indicated.

**Table S2. Chemotherapeutic test of four plant extracts against the growth of *Plasmodium yoelii* in mice**

| **survival rate (%)** | **Mean suppression/group (%)** | **Mice daily parasite growth suppression (%)** | | | | | | **Mean parasitemia of untreated animals** | | **Mice daily parasitemia of treated animals** | | | | | | **Dose of the extract (mg/kg/day)** | | **Plant extract** | | **Dpi** |
| --- | --- | --- | --- | --- | --- | --- | --- | --- | --- | --- | --- | --- | --- | --- | --- | --- | --- | --- | --- | --- |
|  |  | M5 | M4 | M3 | M2 | M1 |  | | M5 | | M4 | M3 | M2 | M1 |  | |  | |  | |
| 100 | 50.3 | 44.8 | 50.3 | 30.9 | 64.1 | 61.3 | 3.6 | | 2.0 | | 1.8 | 2.5 | 1.3 | 1.4 | 100 | | *Artemisia judaica L.* | | 1 | |
| 100 | 13.5 | 36.5 | 1.6 | -6.6 | -8.6 | 44.7 | 4.9 | | 3.1 | | 4.8 | 5.2 | 5.3 | 2.7 |  |  |  |  | 2 | |
| 100 | 43.8 | 48.7 | 24.2 | 63.3 | -0.2 | 82.8 | 8.2 | | 4.2 | | 6.2 | 3.0 | 8.2 | 1.4 |  |  |  |  | 3 | |
| 100 | 56.4 | 43.3 | 56.5 | 62.4 | 49.7 | 70.2 | 20.5 | | 11.6 | | 8.9 | 7.7 | 10.3 | 6.1 |  |  |  |  | 4 | |
| 100 | 60.7 | 58.9 | 49.9 | 76.0 | 49.9 | 68.6 | 24.6 | | 10.1 | | 12.3 | 5.9 | 12.3 | 7.7 |  |  |  |  | 5 | |
| 100 | 45.8 | 73.9 | 75.6 | 21.4 | -1.0 | 59.3 | 24.6 | | 6.4 | | 6.0 | 19.3 | 24.8 | 10.0 |  |  |  |  | 6 | |
| 100 | 53.6 | 45.6 | 51.7 | 53.6 | 72.2 | 45.1 | 41.0 | | 22.3 | | 19.8 | 19.0 | 11.4 | 22.5 |  |  |  |  | 7 | |
| 100 | 17.1 | -24.3 | 19.9 | 53.0 | 22.7 | 14.4 | 3.6 | | 4.5 | | 2.9 | 1.7 | 2.8 | 3.1 | 100 | | *Cleome*  *droserifolla (Forssk.) Delile* | | 1 | |
| 100 | 18.9 | 36.5 | 3.7 | -0.4 | 28.3 | 26.2 | 4.9 | | 3.1 | | 4.7 | 4.9 | 3.5 | 3.6 |  |  |  |  | 2 | |
| 100 | 40.1 | 21.8 | 51.1 | 75.6 | 9.5 | 42.5 | 8.2 | | 6.4 | | 4.0 | 2 | 7.4 | 4.7 |  |  |  |  | 3 | |
| 100 | 49.7 | 14.0 | 36.5 | 67.7 | 53.1 | 77.0 | 20.5 | | 17.6 | | 13.0 | 6.6 | 9.6 | 4.7 |  |  |  |  | 4 | |
| 100 | 38.7 | 60.9 | 38.1 | 46.3 | -3.4 | 51.5 | 24.6 | | 9.6 | | 15.2 | 13.2 | 25.4 | 11.9 |  |  |  |  | 5 | |
| 100 | 24.3 | 45.0 | -6.7 | 23.5 | -5.5 | 65.0 | 24.6 | | 13.5 | | 26.2 | 18.8 | 25.9 | 8.6 |  |  |  |  | 6 | |
| 100 | 61.9 | 67.1 | 45.8 | 74.6 | 56.6 | 65.3 | 41.0 | | 13.5 | | 22.2 | 10.4 | 17.8 | 14.2 |  |  |  |  | 7 | |
| 100 | 65.6 | 61.0 | 86.2 | 70.2 | 49.5 | 61.0 | 4.4 | | 1.7 | | 0.6 | 1.3 | 2.2 | 1.7 | 100 | | *Trichodesma africanum*  *(L.) sm.* | | 1 | |
| 100 | 53.0 | 23.9 | 85.8 | 75.5 | 29.9 | 50.0 | 18.4 | | 14.0 | | 2.6 | 4.5 | 12.9 | 9.2 |  |  |  |  | 2 | |
| 100 | 62.1 | 59.1 | 76.1 | 51.2 | 60.7 | 63.4 | 18.8 | | 7.7 | | 4.5 | 9.2 | 7.4 | 6.9 |  |  |  |  | 3 | |
| 100 | 35.3 | 49.4 | 51.9 | 47.2 | 15.9 | 11.9 | 27.5 | | 13.9 | | 13.2 | 14.5 | 23.1 | 24.2 |  |  |  |  | 4 | |
| 100 | 57.0 | 68.8 | 76.3 | 14.2 | 66.6 | 59.4 | 27.8 | | 8.7 | | 6.6 | 23.9 | 9.3 | 11.3 |  |  |  |  | 5 | |
| 100 | 61.8 | 81.8 | 10.9 | 71.1 | 79.0 | 66.4 | 31.9 | | 5.8 | | 28.4 | 9.2 | 6.7 | 10.7 |  |  |  |  | 6 | |
| 100 | 58.5 | 72.6 | 70.9 | 59.3 | 45.7 | 44.0 | 40.5 | | 11.1 | | 11.8 | 16.5 | 22.0 | 22.7 |  |  |  |  | 7 | |
| 100 | 43.1 | 47.2 | 35.8 | 40.4 | 38.1 | 54.1 | 4.4 | | 2.3 | | 2.8 | 2.6 | 2.7 | 2.0 | 100 | | *Vachellia toritilis*  *subsp. raddiana (Savi) Brenan* | | 1 | |
| 100 | 72.5 | 76.6 | 78.3 | 73.9 | 84.8 | 48.9 | 18.4 | | 4.3 | | 4.0 | 4.8 | 2.8 | 9.4 |  |  |  |  | 2 | |
| 100 | 36.3 | -11.5 | 59.1 | 43.7 | 44.3 | 45.9 | 18.8 | | 21.0 | | 7.7 | 10.6 | 10.5 | 10.2 |  |  |  |  | 3 | |
| 100 | 51.3 | 53.8 | 43.9 | 58.5 | 40.3 | 60.3 | 27.5 | | 12.7 | | 15.4 | 11.4 | 16.4 | 10.9 |  |  |  |  | 4 | |
| 100 | 37.9 | 32.8 | 3.7 | 54.4 | 52.9 | 45.8 | 27.8 | | 18.7 | | 26.8 | 12.7 | 13.1 | 15.1 |  |  |  |  | 5 | |
| 100 | 50.6 | 30.0 | 33.8 | 83.4 | 59.5 | 46.3 | 31.9 | | 22.3 | | 21.1 | 5.3 | 12.9 | 17.1 |  |  |  |  | 6 | |
| 100 | 34.9 | 64.2 | 44.2 | 30.2 | 14.9 | 21.0 | 40.5 | | 13.5 | | 22.6 | 28.3 | 34.5 | 32.0 |  |  |  |  | 7 | |

Estimated daily suppression of *P. yoelii* growth in mice. Parasite growth inhibition percentages were calculated from 3 h post-challenge until the end of the course of treatment at 6 days post-infection for each individual animal against the untreated group mice. All mice were challenged with an intraperitoneal injection of 1×10^7^ *P. yoelii*-infected erythrocytes and then treated with 100 mg/kg/day of each plant extract, administered orally, for 1 week (N = 5 per group). Giemsa-stained thin blood smears were made daily from each mouse, and 600–1,000 RBCs were counted per blood smear. *A. judaica* and *C. droserifolia* extracts were tested in one independent experiment with the same control, and *T. africanum* and *V. tortilis* extracts were tested in another independent experiment sharing the same control. Mean parasite suppression values for all tested plant extracts are illustrated in Figure 2. The percentage of parasitemia was calculated by using the following equation: parasitemia % = [(number of infected RBCs) / (total number of RBCs) ×100]. Daily parasite suppression was calculated by using the following equation: [(mean parasitemia of the untreated group − the parasitemia of the treated group) / (mean parasitemia of the untreated group) × 100]. Dpi, days post-infection.


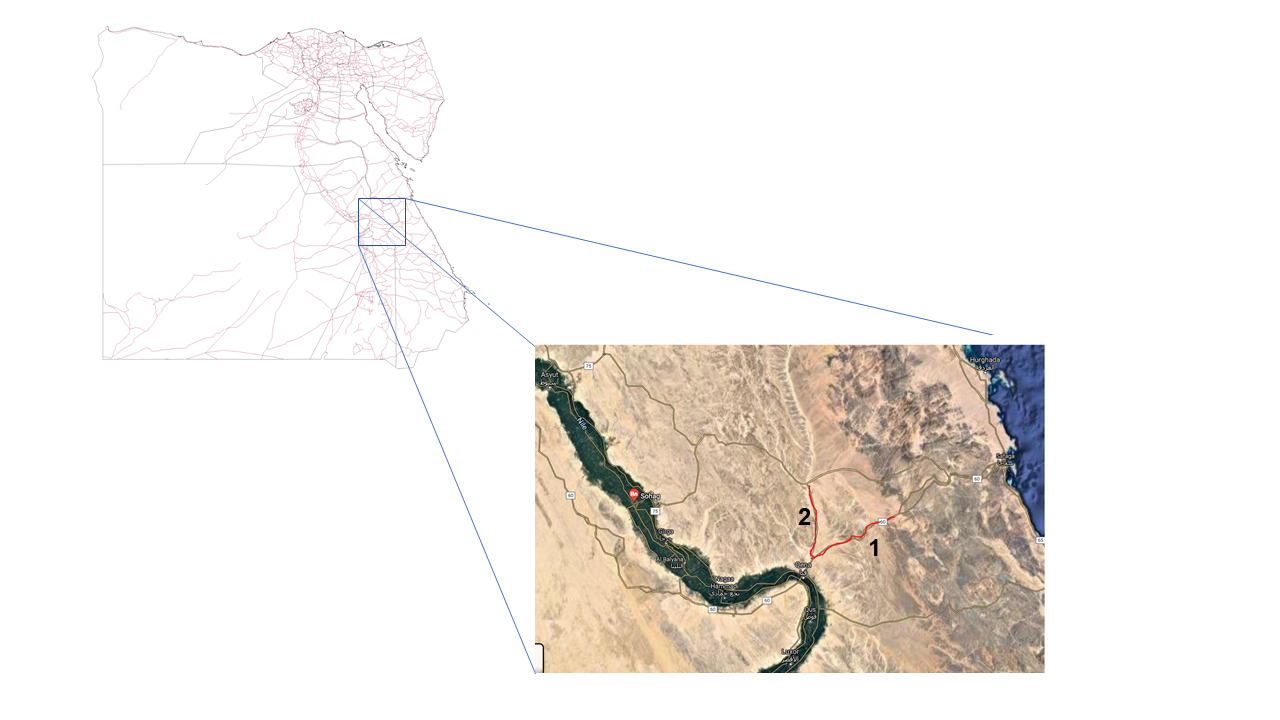


**Figure S1. Sampling map of the plant samples collected in Egypt.** Plant materials were collected during the period from 14–19 May, 2019 in a field survey from two sites in the southern part of Egypt in Qena Governorate. The first site was Qena-Safaga desert road **(1)**, and the second site was Qena-Sohag desert road **(2)**. Plants collection sites coordinates were shown in table S1. Map designed by DIVA-GIS 7.5.0 software to show all country roads and Governorates; the sample collection sites were approximately determined and are shown in the magnified box as highlighted red lines on a Google map.


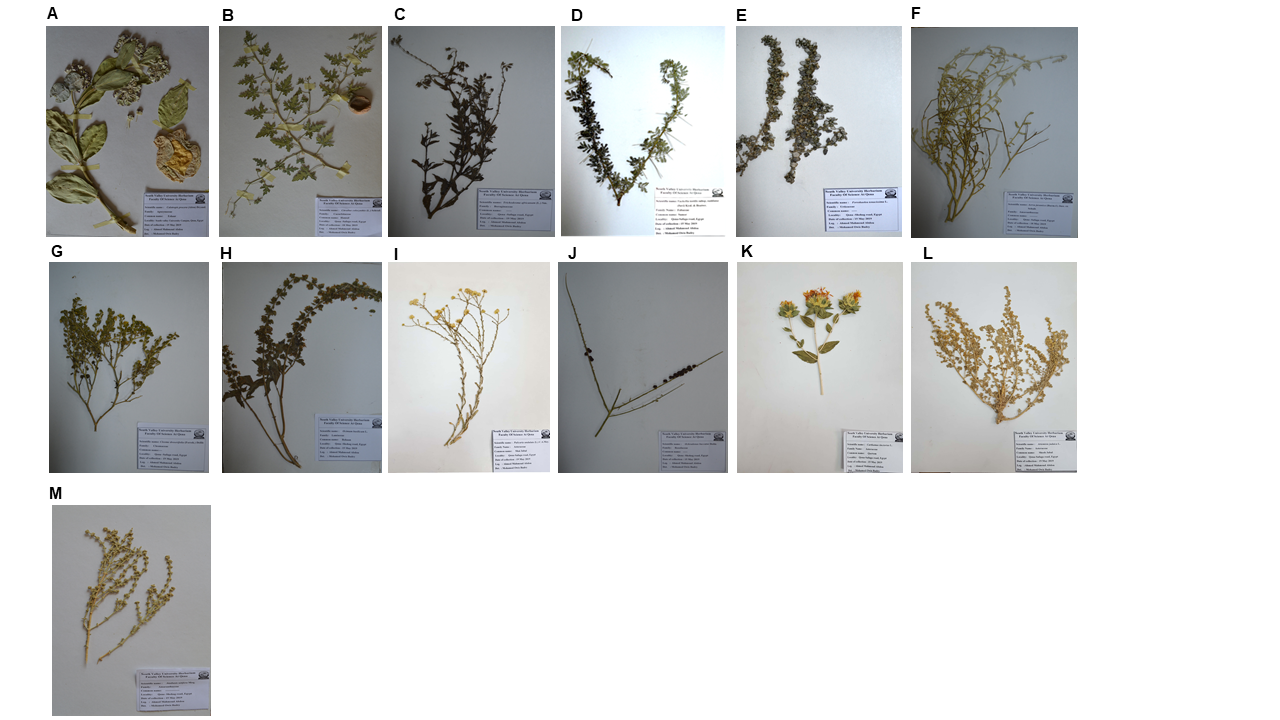


**Figure S2.** **Images of the collected plant materials.** Plants were collected during the period from 14–19 May, 2019. Images of *Calotropis procera* (Aiton) Dryand. **(A)**, Citrullus*colocynthis*(L.) Schrad. **(B)**, *Trichodesma africanum (L.) Sm*. **(C)**, *Vachellia tortilis* subsp.*raddiana*(Savi) Brenan (Aiton) Dryand. **(D)**, *Forsskaolea tenacissima L.* **(E)**, *Aerva javanica* (*Burm.f.*) *Juss. ex Schult.* **(F)**, *Cleome droserifolia* (Forssk.) Delile. **(G)**, *Ocimum basilicum* L. **(H)**, *Pulicaria undulata* (*L*.) *C.A.Mey.* **(I)**, *Ochradenus baccatus* Delile. **(J)**, *Carthamus tinctorius* L. **(K)**, *Artemisia judaica* L. **(L)**, and *Anabasis setifera* Moq. **(M)**. A sample from each collected sample was transferred to the herbarium of the Faculty of Science, South Valley University, Qena, Egypt for identification via microscopy.

**
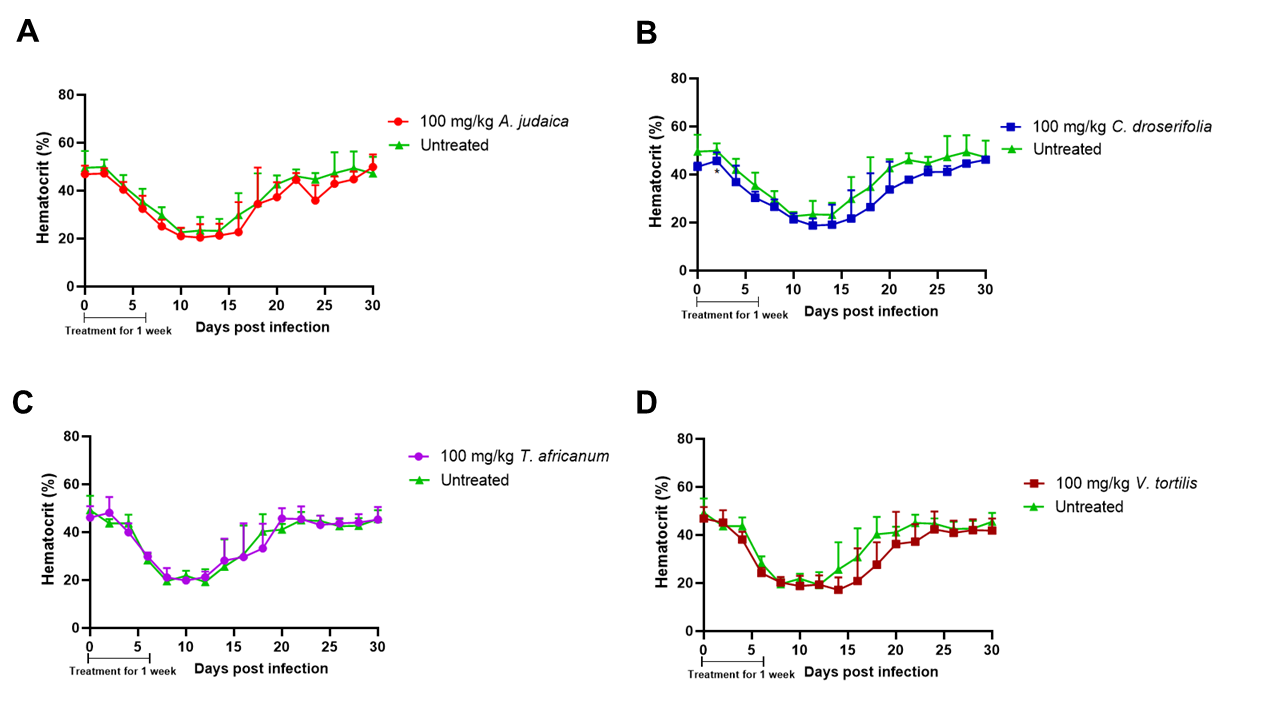
**

**Figure S3. Effect of wild plant extracts on the growth of *Plasmodium yoelii* in male BALB/c mice.** Five mice were used per group. *Artemisia judaica* and *Cleome droserifolia* plant extracts were tested in one independent experiment that shared the same control, and *Trichodesma africanum* and *Vachellia tortilis* plant extracts were tested in another independent experiment that shared the same control. Hematocrit (%) was monitored every other day from day 0 (challenge infection day) until 30 days post-infection. All mice were challenged by an intraperitoneal injection of 1×10^7^ *P. yoelii*-infected erythrocytes and then treated orally with 100 mg/kg/day of one plant extract for 1 week. The untreated group received only PBS. **(A–D)** Hematocrit (%) for the *P. yoelii*-infected mice treated with *A. judaica* **(A)**, C. *droserifolia* **(B)**, *T. africanum* **(C)**, or *V. tortilis* **(D)** extract. Data were analyzed by performing a two-way ANOVA followed by a Bonferroni test against the untreated group (**p* < 0.05).

**
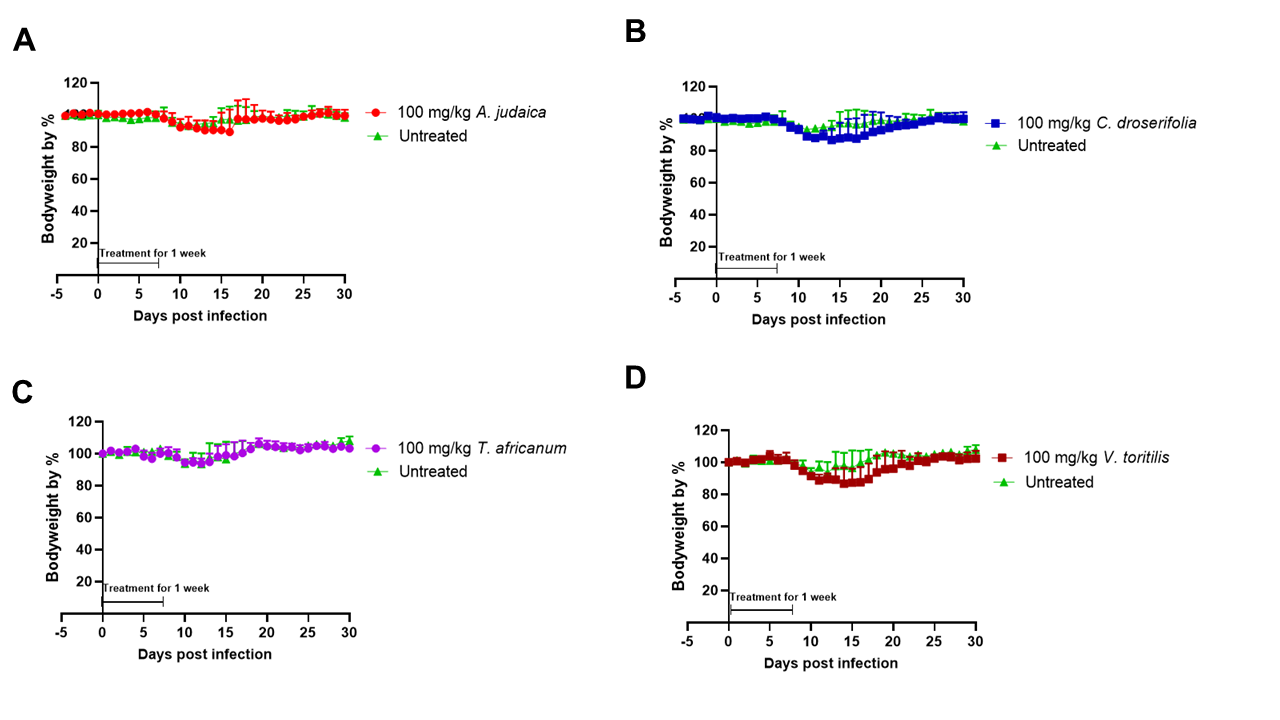
**

**Figure S4.** **Effect of wild plant extracts on bodyweight change in *Plasmodium*-infected mice.** Five male BALB/c mice were used per group. *Artemisia judaica* and *Cleome droserifolia* plant extracts were tested in one independent experiment that shared the same control, and *Trichodesma africanum* and *Vachellia tortilis* plant extracts were tested in another independent experiment that shared the same control. Bodyweight change (%) was monitored daily from day 0 (challenge infection day) until 30 days post-infection. All mice were challenged by an intraperitoneal injection of 1×10^7^ *P. yoelii*-infected erythrocytes and then treated orally with 100 mg/kg/day of one plant extract for 1 week. The untreated group received only PBS. **(A–D)** Bodyweight changes of the *P*. *yoelii*-infected mice treated with *A. judaica* **(A)**, C. *droserifolia* **(B)**, *T. africanum* **(C)**, or *V. tortilis* **(D)** extract. Data were analyzed by performing a two-way ANOVA followed by a Bonferroni test against the untreated group (**p* < 0.05).


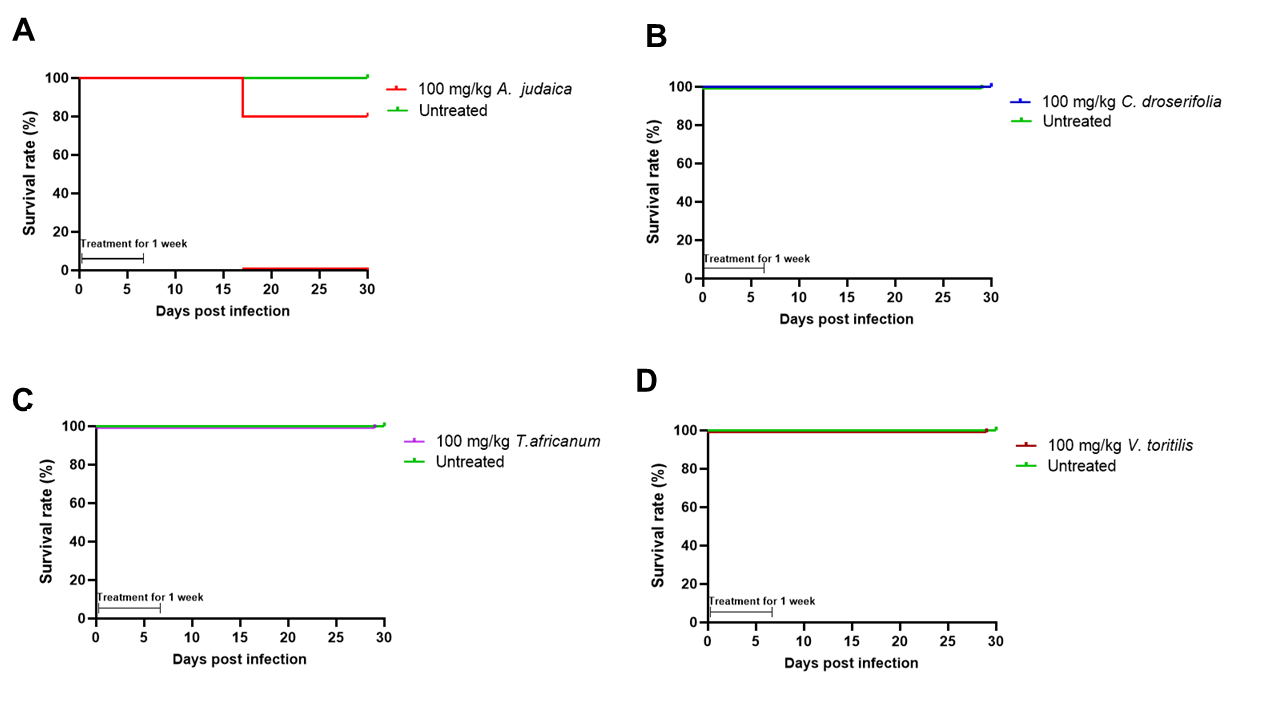


**Figure S5. Effect of wild plant extracts on the survival rate of *Plasmodium*-infected mice**. Five male BALB/c mice were used per group. *Artemisia judaica* and *Cleome droserifolia* plant extracts were tested in one independent experiment that shared the same control, and *Trichodesma africanum* and *Vachellia tortilis* plant extracts were tested in another independent experiment that shared the same control. Survival rates of mice were monitored every day from day 0 (challenge infection day) until 30 days post-infection. All mice were challenged by an intraperitoneal injection of 1×10^7^ *P. yoelii*-infected erythrocytes and then treated orally with 100 mg/kg/day of one plant extract for 1 week. The untreated group received only PBS. **(A–D)** Survival rates of the *P. yoelii*-infected mice treated with *A. judaica* (4/5, 80%) **(A)**, C. *droserifolia* (5/5,100%) **(B)**, *T. africanum* (5/5, 100%) **(C)**, or *V. tortilis* (5/5, 100%) **(D)** extract. In both experiments, the uninfected mice had a 100% survival rate (5/5). The significance of each difference was analyzed with a χ^2^ test, but none of the differences were significantly different.

**References for supplementary information**

1. Movaliya V, Zaveri M. **A review on the Pashanbheda plant *Aerva javanica***. *Int J Pharm Sci Rev Res*. 2014; **25**:268-75.

2. Abdou AM, Abdallah HM, Mohamed MA, Fawzy GA, Abdel-Naim AB. **A new anti-inflammatory triterpene saponin isolated from *Anabasis setifera****. Arch. Pharm. Res*. 2013; **36**:715-22.

3. Mahmoud M., and Gairola S. **Traditional knowledge and use of medicinal plants in the Eastern Desert of Egypt: a case study from Wadi El-Gemal National Park**. *J Med Plants Stud*. 2013; **1**:10-17.

4. Meena AK, Yadav AK, Niranjan US, Singh B, Nagariya AK, Sharma K, Gaurav A, Sharma S, Rao MM. **A review on *Calotropis procera* Linn and its ethnobotany, phytochemical, pharmacological profile**. *Drug Invent. Today* 2010; **2:** 185-90.

5. Kubmarawa D, Ajoku GA, Enwerem NM, Okorie DA. **Preliminary phytochemical and antimicrobial screening of 50 medicinal plants from Nigeria**. *Afr. J. Biotechnol*. 2007;**6**.

6. Sharma P, Sharma JD. **In-vitro schizonticidal screening of *Calotropis procera***. *Fitoterapia* 2000; **71:** 77-9.

7. Mudi SY, Bukar A. **Anti-plasmodia activity of leaf extracts of *Calotropis procera* Linn**. *Biokemistri* 2011; **23**.

8. Al-Snafi AE. **Antiparasitic effects of medicinal plants (part 1)-A review**. *IOSR J. Pharm*. 2016; **6:** 51-66.

9. Zhou X, Tang L, Xu Y, Zhou G, Wang Z. **Towards a better understanding of medicinal uses of *Carthamus tinctorius* L. in traditional Chinese medicine: a phytochemical and pharmacological review**. *J Ethnopharmacol*. 2014; **151:** 27-43.

10. Gurudeeban S, Satyavani K, Ramanathan T. **Bitter apple (*Citrullus colocynthis*): An overview of chemical composition and biomedical potentials.** *Asian J. Plant Sci*. 2010; **9**: 394.

11. Assaf HK, Nafady AM, Kamel MS. **Investigation of the saponifiable and unsaponifiable matter compositions by GC/MS and the antioxidant hepatoprotective activities of aerial parts of *Forsskaolea tenacissima* Linn**. *PJIDRD*. 2017; **2:** 22-32.

12. Sher AA, Afzal M, Bakht J. **Pharmacological evaluation of different extracts of *Forsskaolea tenacissima***. *Indian J. Pharm. Sci*. 2017; **79:** 257-66.

13. Mothana RA, Al-Musayeib NM, Al-Ajmi MF, Cos P, Maes L. **Evaluation of the in vitro antiplasmodial, antileishmanial, and antitrypanosomal activity of medicinal plants used in Saudi and Yemeni traditional medicine**. *Evid-Based compl Alt*. 2014 ;**2014**.

14. Adigüzel A, Güllüce M, Şengül M, Öğütcü H, Şahin F, Karaman İ. **Antimicrobial effects of *Ocimum basilicum* (Labiatae) extract**. *Turk. J. Biol*. 2005; **29:** 155-60.

15. Fahmi AA, Abdur-Rahman M, Naser AF, Hamed MA, Abd-Alla HI, Shalaby NM, Nasr MI. **Chemical composition and protective role of *Pulicaria undulata* (L.) CA Mey. subsp. undulata against gastric ulcer induced by ethanol in rats**. *Heliyon* 2019 ;**5**: e01359.

16. Rav M, Valizadeh J, Noroozifar M, Khorasani-Motlagh M. **Screening of chemical composition of essential oil, mineral elements and antioxidant activity in *Pulicaria undulata* (L.) CA Mey from Iran**. *J. Med. Plant Res*. 2011; **5:** 2035-40.

17. Moustafa SM, Menshawi BM, Wassel GM, Mahmoud K, Mounier MM. **Screening of some plants in Egypt for their cytotoxicity against four human cancer cell lines***. Int J Pharm Tech Res*. 2014; **6:** 1074-84.

18. Jaradat NA, Zaid AN, Abuzant A, Shawahna R. **Investigation the efficiency of various methods of volatile oil extraction from Trichodesma africanum and their impact on the antioxidant and antimicrobial activities**. J*. Intercult. Ethnopharmacol*. 2016; **5:** 250-56.

19. Maregesi SM, Pieters L, Ngassapa OD, Apers S, Vingerhoets R, Cos P, Berghe DA, Vlietinck AJ. **Screening of some Tanzanian medicinal plants from Bunda district for antibacterial, antifungal and antiviral activities**. *J. Ethnopharmacol*. 2008; **119:** 58-66.
